# Species specificity, surface exposure, protein expression, immunogenicity, and participation in biofilm formation of *Porphyromonas gingivalis *HmuY

**DOI:** 10.1186/1471-2180-10-134

**Published:** 2010-05-04

**Authors:** Teresa Olczak, Halina Wójtowicz, Justyna Ciuraszkiewicz, Mariusz Olczak

**Affiliations:** 1 Laboratory of Biochemistry, Faculty of Biotechnology, University of Wroclaw, Tamka 2, 50-137 Wroclaw, Poland

## Abstract

**Background:**

*Porphyromonas gingivalis *is a major etiological agent of chronic periodontitis. The aim of this study was to examine the species specificity, surface exposure, protein expression, immunogenicity, and participation in biofilm formation of the *P. gingivalis *heme-binding protein HmuY.

**Results:**

HmuY is a unique protein of *P. gingivalis *since only low amino-acid sequence homology has been found to proteins encoded in other species. It is exposed on the cell surface and highly abundant in the outer membrane of the cell, in outer-membrane vesicles, and is released into culture medium in a soluble form. The protein is produced constitutively at low levels in bacteria grown under high-iron/heme conditions and at higher levels in bacteria growing under the low-iron/heme conditions typical of dental plaque. HmuY is immunogenic and elicits high IgG antibody titers in rabbits. It is also engaged in homotypic biofilm formation by *P. gingivalis*. Anti-HmuY antibodies exhibit inhibitory activity against *P. gingivalis *growth and biofilm formation.

**Conclusions:**

Here it is demonstrated that HmuY may play a significant role not only in heme acquisition, but also in biofilm accumulation on abiotic surfaces. The data also suggest that HmuY, as a surface-exposed protein, would be available for recognition by the immune response during chronic periodontitis and the production of anti-HmuY antibodies may inhibit biofilm formation.

## Background

Periodontitis is a complex process affecting tooth-supporting tissues [1]. The pathogenesis of periodontal diseases is largely attributed to localized inflammation, which results from interaction between host and microbial factors [2]. The most common etiological agent of chronic periodontitis is *Porphyromonas gingivalis*, a Gram-negative anaerobic black-pigmented bacterium [3]. On tooth surfaces, *P. gingivalis *is a constituent of the complex multispecies biofilm known as dental plaque, which has properties of other biofilms found in the human body and in the environment. *P. gingivalis *can also colonize the tissues and cells of the gingival epithelium [4]. The bacterium can not only invade, but also accumulate inside gingival epithelial cells [5,6]. Recent evidence demonstrates that the effect of periodontitis might have systemic consequences since the bacterium can spread systemically and locate to other tissues [7-10].

Bacteria living in a biofilm have a physiology different from that of planktonic cells and they generally live under nutrient limitation, including that of iron and heme. The uptake of heme as iron and protoporphyrin IX is an important mechanism by which *P. gingivalis *and other pathogenic bacteria obtain these compounds for their survival and their ability to establish an infection [11,12]. Gram-negative bacteria utilize outer-membrane receptors to acquire heme from host hemoproteins directly or through a hemophore or lipoprotein and then transport the captured heme into the cell. In the case of *P. gingivalis*, one of the systems of heme acquisition consists of HmuR and HmuY proteins [12]. HmuR is an outer-membrane TonB-dependent receptor involved in heme transport through the outer membrane [13-16], whereas HmuY is a heme-binding lipoprotein associated with the outer membrane of the bacterial cell [17-21]. A detailed characterization of the HmuY-heme complex demonstrated that heme, with a midpoint potential of 136 mV, is in a low-spin Fe(III) hexa-coordinate environment [20]. In that report we also identified histidines 134 and 166 as potential heme ligands. Recent crystallographic analysis of the HmuY-heme complex confirmed these data and showed that the protein exhibits a unique structure composed of an all-β fold [21]. Our studies also showed that HmuY may be functional in the form of dimers/tetramers [19,21]. It seems that dimeric HmuY takes up heme and this leads to tetramerization under occlusion of the heme binding sites. Tetrameric HmuY would protect heme from host scavengers and delivered it to HmuR. On the basis of our mutational analysis of HmuY heme ligands [20], an initial step in heme transfer could involve disruption of only one of the two axial histidine ligands, as found for *Serratia marcescens *hemophore HasA [22]. Once bound by HmuR, heme is translocated across the outer membrane into the periplasm with the assistance of TonB and further heme transport requires the presence of binding proteins to escort it across the periplasm to the cytoplasm. This step might be performed by other *hmu *operon proteins, so far not characterized [17,19]. HmuY, especially in the form associated with the outer membrane, may also store heme and protect the bacterial cell from damage induced by free hemin.

It is likely that HmuY lipoprotein may play a role not only in heme acquisition, but also in the host pathogen response. Therefore the aim of this study was to analyze the surface exposure and expression of HmuY protein in *P. gingivalis*. In addition, in this report we examined the participation of HmuY protein in biofilm formation.

## Results and Discussion

### HmuY is a unique *P. gingivalis *protein

**Figure 1:**
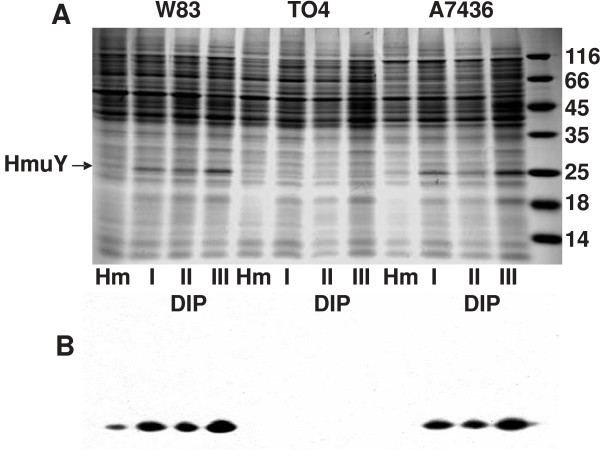
**Analysis of HmuY protein in *P. gingivalis *cell**. Detection of HmuY protein in whole-cell lysates of the wild-type W83 and A7436 strains and the *hmuY *deletion mutant (TO4) strain was performed by SDS-PAGE and Coomassie Brilliant Blue G-250 staining (A) or Western blotting using rabbit anti-HmuY antibodies and chemiluminescence staining (B). Hm, bacteria grown in basal medium supplemented with hemin; DIP, bacteria grown in basal medium supplemented with dipyridyl for the 1^st^, 2^nd^, and 3^rd ^passages.

Preliminary studies demonstrated that HmuY shows high identity to proteins identified in several *P. gingivalis *strains [17,19]. Here we compared the amino-acid sequences of putative HmuY homologues deposited in databases. Interestingly, we found that HmuY is similar to proteins encoded in several different species belonging to the *Bacteroidetes *phylum, which consists of three classes: *Bacteroidetes*, *Flavobacteria*, and *Sphingobacteria *[23]. The *Bacteroidetes *class consists of anaerobes which are often found in high numbers in the intestinal tracts of animals and which may infect different human tissues, including periodontal tissues (see Additional file 1). Members of the other two classes are mainly aerobic and abundant in many freshwater and marine systems (data not shown). Bacteria encoding putative HmuY homologues have also been identified in several human pathogens, including those infecting the oral cavity (see Additional file 1) [24-31]. A characteristic feature of all the HmuY homologues identified in this study is biofilm formation. However, although we found several putative HmuY homologues in a broad range of bacteria, the similarity of the amino-acid sequences of HmuY from *Porphyromonas *and other species was low (5-47%) (see Additional file 1). Only between HmuY proteins encoded within *Porphyromonas *species was the similarity higher (24-100%) (see Additional file 1). In addition, only *P. gingivalis *strains possess both histidines engaged in heme coordination [20,21]. Here we also demonstrated that antibodies against purified HmuY raised in rabbits were highly specific and recognized only this antigen in *P. gingivalis *A7436 and W83 whole-cell lysates compared with a *P. gingivalis hmuY *deletion mutant strain (TO4) (figure 1), *E. coli*, or *Bacteroides fragilis *whole-cell lysates (data not shown).

### HmuY is exposed on the surface of *P. gingivalis *cells

**Figure 2:**
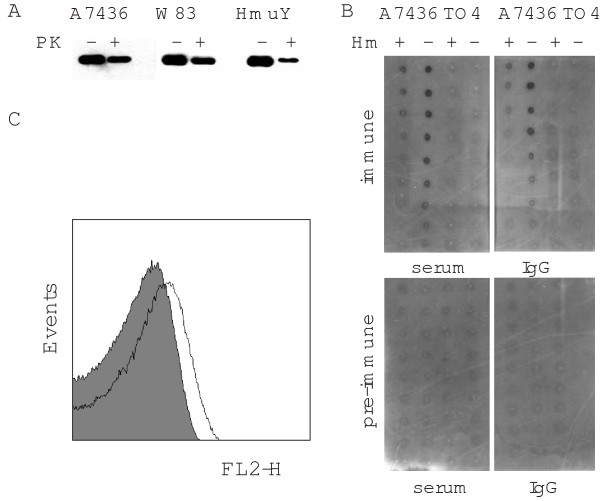
**Analysis of surface exposure of *P. gingivalis *HmuY protein**. (A) Proteinase K (PK) accessibility assay performed with whole-cell *P. gingivalis *wild-type A7436 and W83 strains and the *hmuY *deletion mutant (TO4) grown in basal medium supplemented with dipyridyl and with the purified protein (HmuY). The cells or protein were incubated with proteinase K at 37°C for 30 min and then analyzed by SDS-PAGE and Western blotting. Intact HmuY exposed on the cell surface was analyzed by dot-blotting (B) or FACS (C) analyses. For dot-blotting analysis, varying dilutions of *P. gingivalis *cell suspension (starting at OD_660 _= 1.0; 1 μl) were adsorbed on nitrocellulose membrane and detected with pre-immune serum or purified pre-immune IgGs and immune anti-HmuY serum or purified immune anti-HmuY IgGs. For FACS, *P. gingivalis *cells were washed and, after blocking nonspecific binding sites, incubated with pre-immune (grey) or anti-HmuY immune serum (transparent). Representative data of the *P. gingivalis *A7436 strain are shown.

The N terminus of HmuY shares characteristic features of classical lipoproteins, possessing a signal peptide sequence cleaved off by the signal peptidase II [19,32]. After removal of the signal peptide, the α-amino group of the N-terminal cysteine is acylated, yielding a mature lipoprotein. Although HmuY association with the outer membrane of the *P. gingivalis *cell was previously demonstrated [17,19,33], the orientation of the protein in the outer membrane was not examined. Bacterial lipoproteins may be located at the cell surface or directed into the periplasmic space. We hypothesized previously that HmuY functions as an external protein [21]. To determine whether HmuY is surface exposed, the proteinase K accessibility assay was employed using the *P. gingivalis *A7436 and W83 wild-type strains. Upon incubation with proteinase K of intact cells grown under low-iron/heme conditions, most of the HmuY was not degraded (figure 2A). A similar effect was observed when *P. gingivalis *cells grown under high-iron/heme conditions and *E. coli *cells over-expressing membrane-associated HmuY were examined (data not shown). It is likely that HmuY may be partially protected by the cell wall, similar to other lipoproteins [34], or resistant to proteinase K digestion. The latter is highly possible since we previously demonstrated that HmuY is resistant to the proteolytic action of trypsin and gingipains [21]. Indeed, experiments performed with purified HmuY showed limited degradation of the protein (figure 2A). Therefore we further employed an immunological analysis. Considering the surface-exposed location of HmuY, the protein attached to the *P. gingivalis *cell should be able to react with antibodies. Dot-blotting analysis showed that rabbit anti-HmuY antibodies, either those present in whole immune serum or a purified IgG fraction, recognized surface-exposed HmuY with high affinity compared with pre-immune serum or pre-immune IgGs (figure 2B). We did not detect reactivity with anti-HmuY serum or IgGs in the *hmuY *deletion TO4 mutant cells. A whole-cell ELISA assay highly corroborated that HmuY is associated with the outer membrane and exposed on the extracellular surface of the cell (see Additional file 2). Since these two experiments were performed using adsorbed cells, FACS analysis was employed to examine free cells in solution. The results shown in figure 2C confirmed the surface exposure of HmuY protein. Moreover, all these analyses showed that HmuY is expressed in bacteria grown under low-iron/heme conditions at higher levels than in bacteria grown under high-iron/heme conditions.

### HmuY is one of the dominant proteins produced under low-iron/heme conditions by *P. gingivalis*

**Figure 3:**
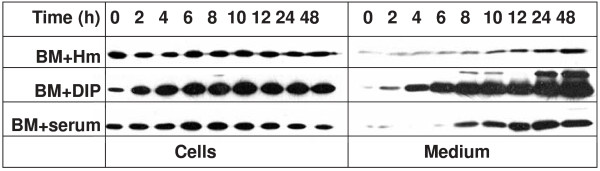
**Determination of HmuY expression in *P. gingivalis *grown under various conditions**. Bacteria (A7436 strain) were grown in basal medium supplemented with hemin (BM+Hm), 160 μM dipyridyl (BM+DIP), or 5% human serum (BM+serum), collected at the indicated time points, centrifuged, and both cells and culture media analyzed by SDS-PAGE and Western blotting with anti-HmuY antibodies.

**Figure 4:**
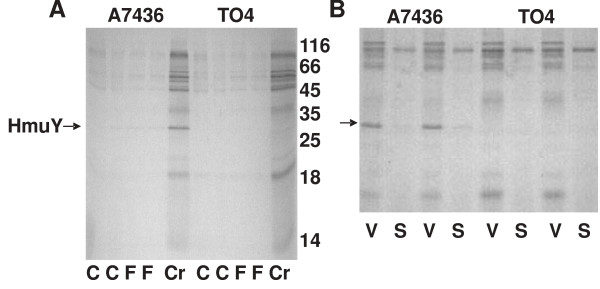
**Analysis of HmuY protein in *P. gingivalis *culture medium**. Detection of HmuY protein in whole culture medium (A) or after fractionation of the culture medium by ultracentrifugation (B) of the wild-type A7436 and the *hmuY *deletion mutant (TO4) strains performed by SDS-PAGE and Coomassie Brilliant Blue G-250 staining. C, culture medium after removal of the cells by centrifugation; F, centrifuged and filtered culture medium; Cr, concentrated culture medium after centrifugation and filtration; V, outer-membrane vesicles; S, soluble proteins present in culture medium after ultracentrifugation.

Previous studies showed that mRNA encoding HmuY was produced at low levels when bacteria were cultured under high-iron/heme conditions (BM supplemented with hemin), but its production was significantly increased when the bacteria were starved in BM without hemin and supplemented with an iron chelator [16,17,19]. To analyze HmuY protein expression in the cell and its release into the culture medium during bacterial growth, Western blotting analysis was employed. We did not detect *P. gingivalis *Fur protein in the culture medium, thus confirming bacterial integrity (data not shown). Similar to mRNA expression, HmuY protein expression was higher in bacteria grown under low-iron/heme conditions (basal medium without added hemin and supplemented with dipyridyl or human serum) than in bacteria grown under high-iron/heme conditions (figures 1 and 3). During bacterial growth, HmuY was constitutively expressed in the cells of the A7436 strain, reaching similar levels in the cells at the indicated time points (figures 3 and 4). Instead of being degraded by active *P. gingivalis *proteases, constitutively produced HmuY was accumulated in the culture medium because during bacterial growth, increasing amounts of the protein were detected in both the outer-membrane vesicle-associated and the soluble form (figures 3 and 4). Our data confirm that the changes observed in gene and protein expression in *P. gingivalis *grown under iron/heme limitation reflect the importance of the environmental levels of these compounds to this bacterium and support the regulation of HmuY expression by iron and heme [19]. The response of *P. gingivalis *to environmental heme availability was previously mapped on a global scale by transcriptomic analysis using DNA microarrays and by proteomic analysis using mass spectrometry [35-37]. The authors found that mRNA levels of *hmuR *and *hmuY *in the cell significantly increased under heme limitation. In contrast to higher levels of HmuR protein produced under heme limitation in the cell, no significant increase in protein levels of HmuY was observed under low-heme conditions. The data presented in this study (figures 1, 3, and 4) and earlier [21] demonstrated that HmuY is constitutively expressed and released into the external milieu not only in the form of outer-membrane vesicles, but also in a soluble form, which precluded the protein from being identified as up-regulated in the proteomic analysis.

In contrast, others have shown that *P. gingivalis *enhanced *hmuY *mRNA expression in response to low cell density rather than to low iron concentration [38]. The authors found that the expressions of the *hmuY *and *hmuR *genes were highest in *P. gingivalis *grown in the early log phase, when the cell density is low, but expression levels were significantly decreased in the late log phase, when cell density is much higher. They also suggested that the expression of *hmuY *mRNA in *P. gingivalis *cells grown in the same cell densities was similar regardless of the presence of heme. These results are different from those demonstrating higher *hmuY *mRNA expression levels in *P. gingivalis *cells grown under low-heme conditions and in biofilm, the latter resembling high-cell-density conditions [35-37]. Our results presented in this study corroborate the latter findings and demonstrate that HmuY protein is constitutively produced in the cell at low levels when bacteria are grown under high-iron/heme conditions; however, significantly higher protein levels are found in cells grown under low-iron/heme conditions, maintained *in vitro *by the addition of an iron chelator or human serum to the heme-free medium (figure 3). These experiments were performed using *P. gingivalis *cultures grown in the first passage of starvation, thus allowing achieving similar cell densities, especially in the early growth phase (data not shown).

### HmuY participates in homotypic biofilm accumulation

**Figure 5:**
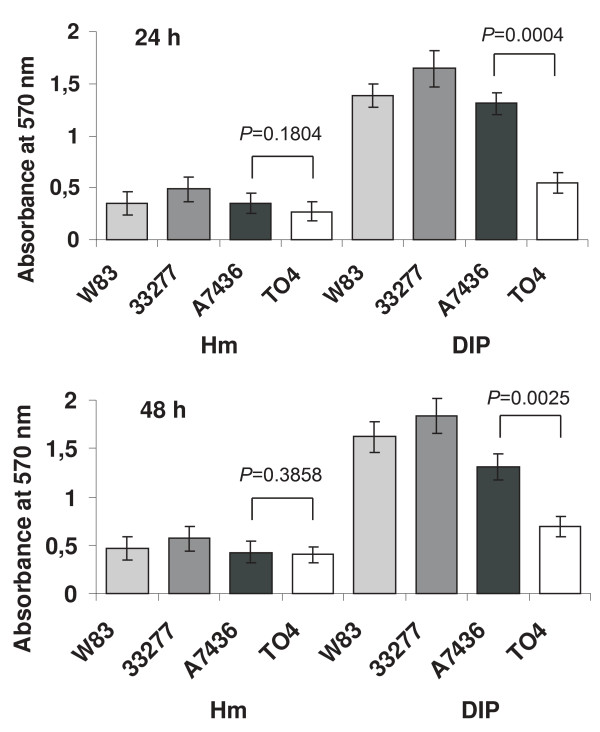
**Homotypic biofilm formation by *P. gingivalis***. *P. gingivalis *wild-type (A7436, W83, and ATCC 33277) strains and the *hmuY *deletion mutant strain constructed in A7436 (TO4) were grown in basal medium supplemented with hemin (Hm) or dipyridyl (DIP). The microtiter plate biofilms were stained with crystal violet. Data are shown as the mean ± *SD *of three independent experiments (n = 24). Differences between the TO4 mutant and the wild-type A7436 strain expressed as *p *values are given above the respective bars.

To cope with a changing environment and with continuous attacks of the host antimicrobial defense systems, bacteria produce a biofilm, which plays an important role in chronic infections due to its ability to challenge the host immune system and resist antimicrobial treatment [39]. It has been demonstrated that *P. gingivalis *actively participates in biofilm formation [40], which facilitates the long-term survival of the bacterium and induces an inflammatory reaction that is responsible for the destruction of the hard and soft tooth-supporting tissues. The transition from planktonic bacteria to biofilm-associated cells involves changes in gene expression and is mediated at least in part by intercellular communication. A recent study demonstrated that HmuY is produced predominantly in *P. gingivalis *cells grown in biofilm compared with the cells growing in a planktonic form [35]. Biofilm formation begins with the production of an extracellular matrix, a structure that creates a shared space within the cellular community. In prokaryotes, the extracellular matrix is typically composed of carbohydrate polymers and proteins, and many of these proteins possess lipoprotein secretion signals. To determine if HmuY could be engaged in biofilm accumulation, we examined *in vitro *the homotypic biofilm-forming capabilities of wild-type (A7436, W83, and ATCC 33277) strains and a *hmuY *deletion mutant constructed in the A7436 strain (TO4). As shown in figure 5, bacteria grown under low-iron/heme conditions exhibited significantly greater biofilm accumulation than cells grown under high-iron/heme conditions. In addition, our data demonstrated that HmuY is involved in biofilm formation since *P. gingivalis *cells not producing this protein showed a significantly lower ability to form biofilm (figure 5). In contrast, all *P. gingivalis *cells grown in a planktonic form exhibited similar growth rates, suggesting that the mutation did not influence bacterial growth (see Additional file 3). All these data suggest that HmuY may play a significant role in biofilm accumulation on abiotic surfaces and support the importance of HmuY for *P. gingivalis *survival during starvation, conditions similar to those found in plaque.

To facilitate adaptation to life within the oral cavity, *P. gingivalis *must be capable of sensing and responding to the prevailing environmental conditions, including nutrient availability, cell density, and the presence of other bacteria. It has been recently shown that *P. gingivalis *possesses the *luxS *gene and produces a functional AI-2 autoinducer [41]. In *P. gingivalis*, among the many different bacterial features that are regulated by quorum sensing using LuxS protein is the expression of genes involved in iron and heme acquisition, including the heme receptor HmuR [41,42]. Although the authors analyzed *hmuR *gene expression only, it is highly possible that the expressions of other components of *hmu *operon, such as *hmuY*, may also be regulated by LuxS signaling. It has been shown that LuxS is also required in *P. gingivalis *for the development of biofilm under low-heme conditions [43], which additionally supports an involvement of HmuY in both heme uptake and biofilm accumulation.

### Anti-HmuY antibodies inhibit *P. gingivalis *growth and biofilm accumulation

**Figure 6:**
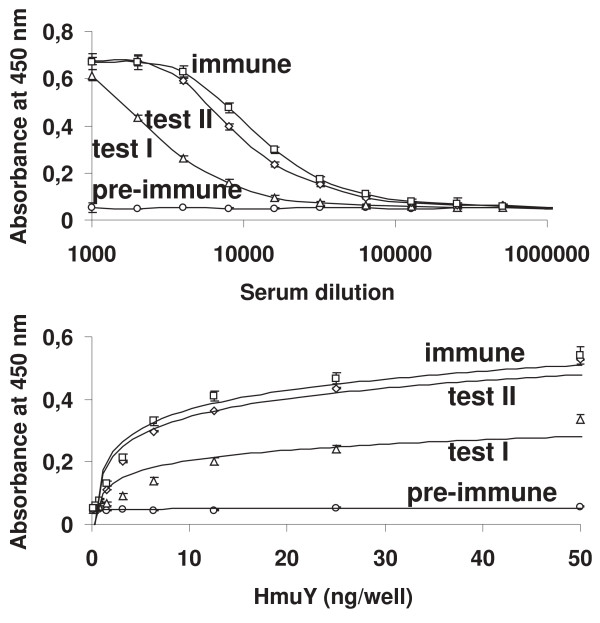
**Production of anti-HmuY antibodies in rabbits**. The reactivity of serial dilutions of rabbit pre-immune and immune anti-HmuY (test I, test II, and immune-serum) sera with 100 ng per well HmuY immobilized on the microtiter plate (A) and the reactivity of pre-immune and immune anti-HmuY (test I, test II, and immune-serum) sera diluted 1:10,000 with varying amounts of HmuY immobilized in the wells of a microtiter plate (B) are shown. Data from three sera analyzed in triplicate are shown as the mean ± *SD*.

**Figure 7:**
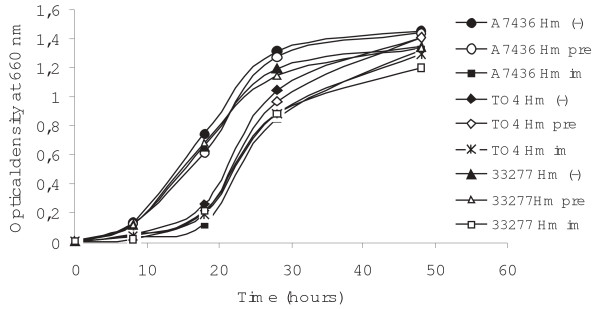
**Inhibition of *P. gingivalis *growth by anti-HmuY IgG antibodies**. The *P. gingivalis *wild-type A7436 and ATCC 33277 strains and the *hmuY *deletion mutant (TO4) strain were grown in basal medium supplemented with dipyridyl. The cells were then washed with PBS, incubated without IgGs (-), with purified pre-immune (pre), or immune (im) anti-HmuY IgGs and inoculated into fresh BM supplemented with hemin (Hm).

**Figure 8:**
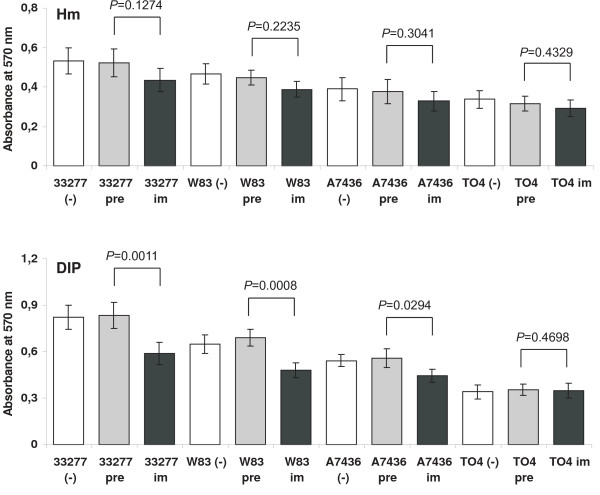
**Inhibition of *P. gingivalis *biofilm formation by anti-HmuY IgG antibodies**. *P. gingivalis *wild-type (A7436, W83, and ATCC 33277) strains and the *hmuY *deletion mutant strain constructed in A7436 (TO4) were grown in basal medium supplemented with hemin (Hm) or dipyridyl (DIP). The cells were washed with PBS, incubated with purified pre-immune or immune anti-HmuY IgGs, and inoculated into fresh media. The microtiter plate biofilms were stained with crystal violet. Data are shown as the mean ± *SD *of three independent experiments (n = 6). Differences between the cells incubated with pre-immune IgGs and cells incubating with immune anti-HmuY IgGs expressed as *p *values are given above the respective bars.

We further tested whether anti-HmuY antibodies had inhibitory activity against *P. gingivalis*, which was first determined by measuring the OD at 660 nm for planktonic bacteria after incubation of bacterial suspensions with pre-immune or immune anti-HmuY IgGs (figure 6). As shown in figure 7, incubation of *P. gingivalis *wild-type strains with immune anti-HmuY IgGs slightly decreased subsequent bacterial growth, especially in the early growth phase. The growth curves resemble those obtained for the *hmuY*-deficient strain. The lack of inhibition of bacterial growth in the late growth phase may be caused by the expression of other iron/heme uptake systems important for *P. gingivalis *at this growth stage. In contrast, anti-HmuY antibodies demonstrated a greater ability to reduce biofilm formation since *P. gingivalis *cells pre-incubated with IgGs isolated from immune anti-HmuY serum exhibited a lower ability of biofilm accumulation than the cells without added IgGs or pre-immune IgGs (figure 8). These data confirm that HmuY protein may be among the proteins important for biofilm accumulation by *P. gingivalis*.

## Conclusions

As the prevalence of antibiotic-resistant strains of bacteria increases, novel ways of treating infections need to be developed. This is particularly important with respect to periodontal diseases, which are the most common chronic bacterial infections of man. First of all, HmuY may be important for a better understanding of the pathology caused by *P. gingivalis*. The surface exposure, high abundance, and immunogenicity of *P. gingivalis *HmuY protein suggest that its detailed examination may yield novel diagnostic methods. Knowledge of the molecular bases of the host immune response against *P. gingivalis *HmuY may be further essential for developing approaches to control and treat chronic periodontitis. To confirm these hypotheses, studies of anti-HmuY antibodies produced in patients with various forms of periodontal diseases and the influence of HmuY and anti-HmuY antibodies on the experimental periodontitis in a mouse model are now underway.

## Methods

### Amino-acid sequence analyses

HmuY homologues were identified using the Basic Local Alignment Search Tool (BLAST; http://blast.ncbi.nlm.nih.gov/Blast.cgi) [44]. Prediction of signal peptides was performed with the LipoP 1.0 Server http://www.cbs.dtu.dk/services/LipoP/[45]. Mature protein sequences were aligned using the CLUSTALW2 program [46] with the default alignment parameters: GONNET 250 protein weight matrix, gap opening penalty 10.00, gap extension penalty 0.2, penalty for closing a gap-1, and penalty for gap separation 4. The phylogenetic tree was constructed with the neighbor-joining method [47]. Bootstrap analysis was performed using 1000 replicates with the CLUSTALW2 program. The tree was drawn with the NJplot program [48].

### Strains and growth conditions

The *P. gingivalis *wild-type strains (A7436, W83, and ATCC 33277), the *hmuY *deletion mutant constructed in the A7436 strain (TO4), and the *Bacteroides fragilis *strain were grown anaerobically on blood agar plates (ABA; Biocorp), in Schaedler broth (Biocorp) and then cultured in basal medium alone (BM), BM supplemented with 1 mg/ml hemin (BM+Hm), 5% human serum (BM+serum), or 160 μM dipyridyl (BM+DIP) as described previously [19]. To avoid autolysis, the bacteria were grown for a time not exceeding 48 h [49]. *E. coli *cells were cultured as indicated in previous reports [18,19].

### HmuY expression and purification

*P. gingivalis *apo-HmuY lacking the first 25 residues (NCBI accession no. CAM 31898) was expressed using pHmuY11 plasmid and *E. coli *ER2566 cells (New England Biolabs) and purified from a soluble fraction of *E. coli *lysate as previously described [19]. The protein concentration was determined as previously reported [20].

### Immunization of rabbits

A non-lipidated form of HmuY (the protein lacking the first 25 amino-acid residues comprising the signal peptide sequence, the following cysteine, and four additional amino acids, GKKK) was used to immunize rabbits (Lampire) with Freund's complete adjuvant. Purified HmuY (0.2 mg per injection) was injected subcutaneously. The animals were boosted on days 7,14, 28, 56, and 84 of the immunization schedule and bled on days 1 (pre-immune serum), 42 (test I serum), 70 (test II serum), and 98 (final-bleed immune serum). The IgG fraction was purified from serum using a HiTrap protein A column according to the manufacturer's instructions (Amersham Pharmacia).

### Protease accessibility assay

To detect HmuY on the surface of the cell, wild-type (A7436, W83), *hmuY*-mutant (TO4), and *E. coli *cells over-expressing membrane-associated HmuY [19] were washed with 20 mM sodium phosphate buffer, pH 7.6, containing 140 mM NaCl (PBS) and re-suspended in 50 mM Tris/HCl, pH 7.6, containing 140 mM NaCl and 10 mM MgCl_2 _to an optical density (OD) of 0.1. The cell suspension was incubated with proteinase K (0.25 mg/ml) for 30 min at 37°C. After incubation, protease inhibitor cocktail (Complete; Roche) was added to stop the reaction, the cells were pelleted, suspended in PBS, and finally the samples were boiled in SDS-PAGE sample buffer. Then the proteins were separated by 15% SDS-PAGE and detected by Western blotting as described below.

### Preparation of cells and proteins for SDS-PAGE and Western blotting

*P. gingivalis *cultures were centrifuged for 30 min at 20,000 × *g *at 4°C and the supernatants were filtered through a 0.22-μm pore-size filter (Roth). Bacterial pellets were washed with PBS and suspended in PBS to OD_660 _= 0.1. To separate outer-membrane vesicles, the filtered culture medium was centrifuged for 2 h at 100,000 × *g*. For HmuY expression analysis, samples corresponding to 5 μl of the bacterial culture at OD_660 _= 0.1 or 20 μl of the culture medium were separated by 15% SDS-PAGE and transferred onto nitrocellulose membranes (Schleicher & Schuell). Nonspecific binding sites were blocked with 5% skim milk in PBS. HmuY was visualized with polyclonal anti-HmuY rabbit serum (Lampire) and secondary goat anti-rabbit IgG antibodies conjugated with horseradish peroxidase (HRP; Sigma), both used at 1:10,000 dilutions. The reaction was developed using chemiluminescence reagents (Western Lightning *Plus*-ECL; Perkin Elmer). To determine *P. gingivalis *autolysis, the presence of Fur was examined in both cells and culture medium using Western blotting with rabbit polyclonal antibodies raised against the synthetic peptide derived from the amino-acid sequence of Fur (CILADKDLRPPRFSY; GeneScript).

### Enzyme-Linked Immunosorbent Assay (ELISA)

Levels of anti-HmuY antibodies in rabbit sera were determined by ELISA. For this purpose, 96-well polystyrene plates (Polysorp; Nunc) were coated for 1 h at 37°C with 100 μl/well HmuY in PBS. The plates were washed three times with 200 μl of PBS prior to blocking for 1 h at 37°C with 200 μl of 2% bovine serum albumin (BSA) dissolved in PBS and then washed three times with 200 μl of PBS. Two-fold serum dilutions or 1:10,000 serum dilutions (100 μl of pre-immune, test I, test II, and immune serum) were prepared in PBS and incubated for 1 h at 37°C. After washing, antibody binding was detected using goat anti-rabbit IgG conjugated with HRP. After three final washes, a substrate solution (100 μl) containing 0.05% *o*-phenylenediamine (Sigma) with 0.01% H_2_O_2 _was added for color development at room temperature. The reaction was stopped after 15 min by adding 25 μl of 12.5% H_2_SO_4 _and the absorbance was measured at 450 nm using a Multiskan Ascent microplate reader (Thermo Electron Corporation).

### Whole-cell ELISA, dot-blotting, and FACS analyses

As an additional method of HmuY detection, cell surface staining with anti-HmuY antibodies was performed using whole-cell ELISA, dot-blotting, and flow cytometry (FACS) analyses. *P. gingivalis *cells grown to OD_660 _= 1.0 were used for these experiments. For the ELISA and dot-blotting analyses, washed cells at several dilutions were adsorbed on the surface of microtiter plates or nitrocellulose membranes. Nonspecific binding of antibodies was prevented by incubation with 1% bovine serum albumin and 2% bovine fetal serum (Sigma) before the addition of rabbit pre-immune or anti-HmuY immune serum (1:10,000) or purified IgG fractions (100 ng/ml). After 1-h incubation and washing with PBS, goat HRP-conjugated (Sigma) or bovine phycoerythrin-conjugated anti-rabbit IgG (Santa Cruz Biotechnology) at 1:10,000 or 1:500 dilutions were used, respectively. Finally, the cells, wells, and membranes were washed with PBS. For FACS analysis, the cells were fixed with 2% *p*-formaldehyde. Then absorbance at 450 nm (ELISA), chemiluminescence (dot-blotting analysis), or fluorescence (FACS; Excalibur, Beckton Dickinson) were detected.

### Biofilm formation

Homotypic biofilm formation by *P. gingivalis *was performed as described by others [50]. Briefly, *P. gingivalis *cells were grown on ABA plates, then in BM supplemented with hemin or dipyridyl to OD_660 _= 1.0 and used to inoculate fresh cultures to OD_660 _= 0.1. Cells in the appropriate medium were transferred (200 μl) into sterile round-bottom microtiter plates (Sarstedt) and incubated under anaerobic conditions at 37°C for 24 or 48 h. The resulting biofilms were washed with PBS, stained with 1% crystal violet, washed with PBS, and de-stained with 96% ethanol. Absorbance (A) was determined at 570 nm using a Multiskan Ascent microplate reader. The assays were repeated at least three times with each strain grown in eight wells. To confirm that the *P. gingivalis *cells were viable, the biofilm cells were scrapped into the respective medium and the OD at 660 nm and colony-forming unit (CFU) values were evaluated after 24 and 48 h (see Additional file 3). In parallel, bacteria were grown in planktonic form and the OD at 660 nm and CFU values were measured after 24 and 48 h.

### Growth and biofilm inhibition studies

Bacteria were grown overnight on ABA plates and then in BM supplemented with hemin or dipyridyl to OD_660 _= 1.0. After centrifugation, the bacteria were washed and suspended in PBS to OD_660 _= 0.1. Then 5 ml of the bacterial suspension was centrifuged and the bacteria were incubated in 200 μl of PBS for 1 h at 37°C with the IgG fraction purified from pre-immune or immune anti-HmuY rabbit serum (200 ng). After addition of 5 ml of the appropriate medium, planktonic bacterial growth was monitored by measuring the OD at 660 nm or biofilm formed as described above. Assays were performed three times in duplicate.

### Statistical analysis

Data are expressed as means values ± standard deviations (mean ± *SD*). Statistical analysis was performed using unpaired Student's *t *test (GraphPad Prism 5). Values of *p *< 0.05 were considered statistically significant.

## Authors' contributions

TO conceived the study, contributed to its design, laboratory experiments, and data analysis and wrote the manuscript. HW, JC, and MO contributed to the design, laboratory experiments, and the writing of the manuscript. All authors have read and approved the final manuscript.

## Supplementary Material

Additional file 1 **Comparison of HmuY homologues**. Comparison of homologous HmuY amino-acid sequences identified in human pathogens (A) and bacteria identified in oral tissues (B). Amino-acid sequences lacking signal peptides are shown. Positions with identical amino acids in more than 30% of the sequences are shown in black boxes and partial homology is indicated in grey boxes. Phylogenetic relationship between homologous HmuY amino-acid sequences (C). Bacteria infecting the oral cavity are shown in bold. The phylogenetic tree was determined with the Neighbor-Joining method. Bootstrap values are included. Pgi, *Porphyromonas gingivalis*; Pen, *P. endodontalis*; Pue, *P. uenonis*; Bfr, *Bacteroides fragilis*; Bfi, *B. finegoldii*; Bco, *B. coprocola*; Bst, *B. stercoris*; Bdo, *B. dorei*; Bvu, *B. vulgatus*; Bov, *B. ovatus*; Bca, *B. caccae*; Bth, *B. thetaiotaomicron*; Bcp, *B. coprophilus*; Bsp, *Bacteroides *sp.; Coc, *Capnocytophaga ochracea*; Cgi, *C. gingivalis*; Csp, *C. sputigena*; Lbo, *Leptospira borgpetersenii*; Lin, *L. interrogans*; Ssp, *Sphingobacterium spiritivorum*; Pbi, *Prevotella bivia*; Por, *P. oris*; Pbe, *P. bergensis*; Pti, *P. timonensis*; Pme, *P. melaninogenica*; Pve, *P. veroralis*; Psp, *Prevotella *sp.; Pta, *P. tannerae*. Click here for file

Additional file 2 **Analysis of surface exposure of HmuY**. Analysis of surface exposure of *P. gingivalis *HmuY analyzed by whole-cell ELISA. *P. gingivalis *wild-type (A7436, W83) and *hmuY *deletion mutant (TO4) strains were grown in basal medium supplemented with hemin (Hm) or dipyridyl (DIP). The cells were washed and diluted with PBS (starting at OD_660 _= 1.0). Varying dilutions of *P. gingivalis *cells were adsorbed on the wells of the microtiter plate and reacted with pre-immune serum (A) or purified pre-immune IgGs (pre) (B) and immune anti-HmuY serum (A) or purified immune anti-HmuY IgGs (im) (B). Representative data are shown. Click here for file

Additional file 3 ***P. gingivalis *growth in broth cultures and biofilms, and biofilm accumulation**. *P. gingivalis *growth was analyzed by measuring the OD at 660 nm, cell viability by plating cells on ABA plates and colony forming unit (CFU) calculation, and biofilm accumulation by microtiter plate assay. Click here for file
