# Supplementary material for: Species specificity, surface exposure, protein expression, immunogenicity, and participation in biofilm formation of Porphyromonas gingivalis HmuY
Source: BMC Microbiol. 2010 May 4;10:134. doi: 10.1186/1471-2180-10-134 (PMC2873494; doi:10.1186/1471-2180-10-134)
Supplement: Additional file 1 — Comparison of HmuY homologues. Comparison of homologous HmuY amino-acid sequences identified in human pathogens (A) and bacteria identified in oral tissues (B). Amino-acid sequences lacking signal peptides are shown. Positions with identical amino acids in more than 30% of the sequences are shown in black boxes and partial homology is indicated in grey boxes. Phylogenetic relationship between homologous HmuY amino-acid sequences (C). Bacteria infecting the oral cavity are shown in bold. The phylogenetic tree was determined with the Neighbor-Joining method. Bootstrap values are included. Pgi, Porphyromonas gingivalis; Pen, P. endodontalis; Pue, P. uenonis; Bfr, Bacteroides fragilis; Bfi, B. finegoldii; Bco, B. coprocola; Bst, B. stercoris; Bdo, B. dorei; Bvu, B. vulgatus; Bov, B. ovatus; Bca, B. caccae; Bth, B. thetaiotaomicron; Bcp, B. coprophilus; Bsp, Bacteroides sp.; Coc, Capnocytophaga ochracea; Cgi, C. gingivalis; Csp, C. sputigena; Lbo, Leptospira borgpetersenii; Lin, L. interrogans; Ssp, Sphingobacterium spiritivorum; Pbi, Prevotella bivia; Por, P. oris; Pbe, P. bergensis; Pti, P. timonensis; Pme, P. melaninogenica; Pve, P. veroralis; Psp, Prevotella sp.; Pta, P. tannerae. [file 1471-2180-10-134-S1.DOC]

Ssp_EEI93473 ----------------------------CSKSDTPQPDPEPEPPTAESMFNTIITVKNFGEALPQGS------------- 39
Ssp_EER70254 ----------------------------CSKSDTPQPDPEPEPPTAESMFNTLITVKNFGEALPQGS------------- 39
Ssp_EEI91310 ----------------------------CSKSND-GPNVELSDGTSTVIADLAGDVDASVGNSAPGK------------- 38
Ssp_EER72820 ----------------------------CSKSND-GPNVELSDGTSTVITDLAGDVDASVGNSAPGK------------- 38
Bfr_CAH07859 ------------------------------------------CVDYSDIQPFDGKTLPRKSGYTTGV------------- 25
Bsp_EEZ27613 ------------------------------------------CVDYSDIQPFDGKTLPRKSGYTTGV------------- 25
Bco_EDV01216 ---------------------------------------------YDDIQPFTGKTLPRKSGYSTGV------------- 22
Pbi_EFB92697 ------------------------------------------CVSY-DAEEFTGKTLPRVTGYTTGV------------- 24
Por_EFB31294 ------------------------------------------CVKY-DAEPFTGKTLPRVSGYSTGV------------- 24
Bfr_ZP_05280704 ------------------------------------------CVKY-DAEPFTGKTLPRTCGYSTGV------------- 24
Pbe_EFA44395 ------------------------------------------CVHY-DADEFEGKVMPRATGAYTGV------------- 24
Pti_EFA97970 ------------------------------------------------AEEFTGKVMPRVASYTTGV------------- 19
Bfr_CAH06766 ----------------------------------CS-DNNS--PDDPSQGENTLPVKQVSLSRKTAY------------- 30
Bfr_BAD47866 ----------------------------------CS-DNNS--PDDPSQGENTLPVKQVSLSRKTAY------------- 30
Bsp_EES87618 ----------------------------------CS-DNNS--PDDPSQGENTLPVKQVSLSRKTAY------------- 30
Bsp_EEZ26547 ----------------------------------CS-DNNS--PDNPSQGENALPVKQVSLSRKTAY------------- 30
Bfr_ZP_05280033 ----------------------------------CSGDNNGSLPDDPSQGGGALPVKQVSLSRKTAY------------- 33
Pue_EEK17166 ----------------------------------CG--DNK--PNAPSS-KNKKTYTQ-SRRCKGEL------------- 27
Cgi_EEK15165 ------------------------------CSKDSN-------KEEEKK----PANVKQEKNLNAR-------------- 25
Coc_ACU93343 ------------------------------CSKDND-------SKEEKK----ISKGTQVKNLYTFN------------- 26
Cgi_EEK14790 ------------------------------CEKE---------KGEETI----LSVTKDVKMLNATS------------- 24
Csp_EEB66232 ------------------------------CSKD---------DKKTEA----PKLPYKEQKVDASS------------- 24
Pgi_ABL74281 ------------------------------CGKKKD-------EPNQPS---TPEAVTKTVTIDASK------------- 27
Pgi_CAM31898 ------------------------------CGKKKD-------EPNQPS---TPEAVTKTVTIDASK------------- 27
Pgi_AAQ66587 ------------------------------CGKKKD-------EPNQPS---TPEAVTKTVTIDASK------------- 27
Pgi_CAM31897 ------------------------------CGKKKD-------EPNQPS---TPEAVTKTVTIDASK------------- 27
Pgi_BAG33077 ------------------------------CGKKKD-------EPNQPS---TPEAVTKTVTIDASK------------- 27
Pen_EEN82265 -----------------------MVLGVASCRPNQTPKPGPTPTPNPPA---QEEVKGLKGYVDASK------------- 41
Pue_EEK17583 ------------------------------CHKPAPQPGHTPDDPVTPPADPKKEWMVKRMTIDATD------------- 37
Pue_EEK16193 ------------------------------CKSDKNEP-----KPVPNPSQEKG--MTKKIVVDASD------------- 30
Bsp_EEO56990 ------------------------------CNGIFENIYD---APIET--------EMEIKENSFS---QVKTV------ 30
Bsp_ZP_0561712 ------------------------------CNGIFENIYD---APIET--------EMEIKENSFS---QVKTV------ 30
Bov_EDO10376 ------------------------------CNGIFENIYD---APIET--------EMEIKENSFS---QVKTV------ 30
Bca_EDM22026 ------------------------------CNGLFENIYD---APTET--------EMEIKENSFS---QIKTV------ 30
Bsp_EES70848 ------------------------------CNGMFEGIYD---DPIEA--------EMEIKESSFS---QINAT------ 30
Bth_AAO75604 ------------------------------CNGMFEGIYD---DPIEA--------EMEIKESSFS---QINAT------ 30
Bdo_EEB24050 ------------------------------CDGILEGIYD---SPAAS--DSNEFGFIRTDPSTHSGTIYIDAT------ 39
Bsp_EEZ23549 ------------------------------CDGILEGIYD---SPAAS--DSNEFGFIRTDPSTHSGTIYIDAT------ 39
Bsp_EEO61578 ------------------------------CDGILEGIYD---SPAAS--DSNEFGFIRTDPSTHSGTIYIDAT------ 39
Bsp_EEO47827 ------------------------------CDGILEGIYD---SPAAS--DSNEFGFIRTDPSTHSGTIYIDAT------ 39
Bsp_EET16222 ------------------------------CDGILEGIYD---SPAAS--DSNELGFIRTDPSTHSGTIYIDAT------ 39
Bvu_ABR39853 ------------------------------CDGILEGIYD---SPAAS--DSNELGFIRTDPSTHSGTIYIDAT------ 39
Bsp_EEZ28283 --------------------------------GILSSLYD---EPET----AKDFGFITIDHANHSGTVRVDAT------ 35
Bsp_EES84662 --------------------------------GILSSLYD---EPET----AKDFGFITIDHANHSGTVRVDAT------ 35
Bfr_CAH08403 --------------------------------GILSSLYD---EPET----AKDFGFITIDHANHSGTVRVDAT------ 35
Bfr_BAD49437 --------------------------------GILSSLYD---EPET----AKDFGFITIDHANHSGTVRVDAT------ 35
Bfr_ZP_05281773 ------------------------------CNGILSNIYD---EPET----AKDFGFIAIDRANHSGTVRVDAT------ 37
Bcp_EEF75672 MTTSNYPLMKKYRFHVKAAMLGTLLLTFPACEGLFDDIYD---NPSEANLSTNGFGFVEVSPETHSGTLYVNSS------ 71
Por_EFB31210 -----------------------MTGTIVLSFLSCDNIYD---DPSEG------DPSQHKKDNTYT---NINAT------ 39
Pme_EES82771 ------------------------------CNGIFEDIYD---EAPAT-ANVTTEGQLLVNAASWKDWYYVDFDSLQMYI 46
Pve_EEX17867 ------------------------------CNGLFDGIYD---DAPAS-PTIT-EGQLLVDATSWKDWYYVDFDSLQAYI 45
Pta_EEX72705 ------------------------------CNGILGGIYD---EQPDK-AAVA-PGTLYIDASGWTDWHYVDLDSLAALQ 45
Psp_EEX53869 ------------------------------CTGLFDGIYD---SPDKA-PKIN-ANQLAIDASDWHNWYYIDFDSLQMLA 45
Bfi_EEX45726 ------------------------------CSDDD--------EKK----EETEMKTLTVDATAYNQWVYVNLKD----- 33
Bst_EDS13961 ------------------------------CSDDD--------NKGSQWGDGVGGTKTNLDVSAYDKWTYVNLKT----- 37
Pta_EEX71812 ------------------------------CSKDDPAPKPNP-KPDPNPEVPAQVKQITVTATEYDTWTYVDLKT----- 44
Por_EFB32073 ------------------------------CSKDDNTPVN----PQNVNSKRLTLNASPR-----DSWLYVNLET----- 36
Pti_EFA97464 ------------------------------CDKSEDLSQKKEKEEQSVICKHADILLQEKQMTNYDKWVYIDLES----- 45
Lbo_ABJ77549 ------------------------------CARQHSAVDN----EELAFKQSILALQKQIEEANRSKILSTELNE----- 41
Lbo_ABJ80492 ------------------------------CARQHSAVDN----EELAFKQSILALQKQIEEANRSKILSTELNE----- 41
Lin_AAS72180 ---------------------------LFQCARQHPAVDQ----NEVAFQQALLDLQKQIEESTRSKIISTEPNG----- 44

Ssp_EEI93473 -----------------------------------EPTTKQSPIYFSLESKQ-----------GINPDYKQT-------- 65
Ssp_EER70254 -----------------------------------EPTTKQSPIYFSLESKQ-----------GINPDYKQT-------- 65
Ssp_EEI91310 -----------------------------------EKRDFHTFLFRFSDQKQTWL-----KTAADSAKYIKG-------- 70
Ssp_EER72820 -----------------------------------EKRDFHTFLFRLSDQKQTWL-----KTAADSAKYIKG-------- 70
Bfr_CAH07859 -----------------------------------TNDWIYFNLRTGEIFNALGV-----NRDIKEGGQMNR-------- 57
Bsp_EEZ27613 -----------------------------------TNDWIYFNLRTGEIFNALGV-----NRDIKEGGQMNR-------- 57
Bco_EDV01216 -----------------------------------TNDWIYFNLRTGESFNTYTV-----NKDIKEGEQINR-------- 54
Pbi_EFB92697 -----------------------------------TNDWLYINLRTGKIFNLDKP-----NGDIKEGEQRER-------- 56
Por_EFB31294 -----------------------------------TNDWIYFNLRTGEVFNSYQP-----GSDIKEGEQRNR-------- 56
Bfr_ZP_05280704 -----------------------------------TNDWIYFNLRTGERFNTLSP-----NQDITEGEQKER-------- 56
Pbe_EFA44395 -----------------------------------TDDWLYFNLRTGKAFNLSAP-----NQDITEGDQLKR-------- 56
Pti_EFA97970 -----------------------------------TNDWIYFNLRTGEIFNRTAP-----NRDIREGQQQSR-------- 51
Bfr_CAH06766 -----------------------------------GNDWIYYSLEKGKEVS------------VSEESHAEN-------- 55
Bfr_BAD47866 -----------------------------------GNDWIYYSLEKGKEVS------------VSEESHAEN-------- 55
Bsp_EES87618 -----------------------------------GNDWIYYSLEKGKEVS------------VSEESHAEN-------- 55
Bsp_EEZ26547 -----------------------------------GNDWIYYSLEKGKEVS------------VSEESHAEN-------- 55
Bfr_ZP_05280033 -----------------------------------GNDWIYYSLEKGKEVN------------ISEEAHAEN-------- 58
Pue_EEK17166 -----------------------------------GKDWIYFSFATGKEVPG-----------IDETNFKER-------- 53
Cgi_EEK15165 -----------------------------------QEKWVYYSFEKNAIVEVA--------------DPQNS-------- 48
Coc_ACU93343 -----------------------------------KDNWVYFSFKK-GVITIQ--------------DPENS-------- 48
Cgi_EEK14790 -----------------------------------YQKWVYYSFEKGAVVEVS--------------SPETD-------- 47
Csp_EEB66232 -----------------------------------YDKWVYFSFENGVVTSTT--------------ATPTT-------- 47
Pgi_ABL74281 -----------------------------------YETWQYFSFSKGEVVNVT--------------DYKND-------- 50
Pgi_CAM31898 -----------------------------------YETWQYFSFSKGEVVNVT--------------DYKND-------- 50
Pgi_AAQ66587 -----------------------------------YETWQYFSFSKGEVVNVT--------------DYKND-------- 50
Pgi_CAM31897 -----------------------------------YETWQYFSFSKGEVVNVT--------------DYKND-------- 50
Pgi_BAG33077 -----------------------------------YETWQYFSFSKGEVVNVT--------------DYKND-------- 50
Pen_EEN82265 -----------------------------------YEKWVYYSLKDNKEVEVS--------------DYKNS-------- 64
Pue_EEK17583 -----------------------------------YTKWVYLNFTTGELVQVT--------------DPAND-------- 60
Pue_EEK16193 -----------------------------------YTKWVYINFDKGEVVSVS--------------TPETD-------- 53
Bsp_EEO56990 ----------------------------------EYTEWAYINLSERTVTTVKI-----------GEEYESQ-------- 57
Bsp_ZP_0561712 ----------------------------------EYTEWAYINLSERTVTTVKI-----------GEEYESQ-------- 57
Bov_EDO10376 ----------------------------------EYTEWAYINLSERTVTTVKI-----------GEEYESQ-------- 57
Bca_EDM22026 ----------------------------------EYTEWAYIDFSGRKVTTVKI-----------GKEYESE-------- 57
Bsp_EES70848 ----------------------------------EYTNWVYIDLSERKATTVEI-----------GEEHKSE-------- 57
Bth_AAO75604 ----------------------------------EYTNWVYIDLSERKATTVEI-----------GEEHKSE-------- 57
Bdo_EEB24050 ----------------------------------DYRRWTFIDFHTQKVDSVNV-----------TDSDQKE-------- 66
Bsp_EEZ23549 ----------------------------------DYRRWTFIDFHTQKVDSVNV-----------TDSDQKE-------- 66
Bsp_EEO61578 ----------------------------------DYRRWTFIDFHTQKVDSVNV-----------TDSDQKE-------- 66
Bsp_EEO47827 ----------------------------------DYRRWTFIDFHTQKVDSVNV-----------TDSDQKE-------- 66
Bsp_EET16222 ----------------------------------DYRRWTFIDFHTQKVDSVNV-----------TDSEQKE-------- 66
Bvu_ABR39853 ----------------------------------DYRRWTFIDFHTQKVDSVNV-----------TDSEQKE-------- 66
Bsp_EEZ28283 ----------------------------------QYTKWNYINLHTLQIDSAKV-----------TAEGADD-------- 62
Bsp_EES84662 ----------------------------------QYTKWNYINLHTLQIDSAKV-----------TAEGADD-------- 62
Bfr_CAH08403 ----------------------------------QYTKWNYINLHTLQIDSAKV-----------TAEGADD-------- 62
Bfr_BAD49437 ----------------------------------QYTKWNYINLHTLQIDSAKV-----------TAEGADD-------- 62
Bfr_ZP_05281773 ----------------------------------QYTKWNYINLHTLRIDSAKI-----------TSEGAED-------- 64
Bcp_EEF75672 ----------------------------------DYTQWVYIDLHSLSVDSTRI-----------LNEAGEEVSLSDKGT 106
Por_EFB31210 ----------------------------------EYTNWVYLNLKNG--SQKTL-----------LYDNTAD-------- 64
Pme_EES82771 ERKDTAGLLKAQTNFTHYAIPTNLTSGSGDGKTGMYTYWFDVFGKGISVNEKRG-----------FTSTDAQ-------P 108
Pve_EEX17867 EKKDTVGLLKAQSHFTPYHIPSSLSTGTSNGQTGIYTYWFDVFGKGIAVNEKRS-----------FKVTDAQ-------P 107
Pta_EEX72705 AAGDSVGLRRAQTHFTHFPIPT--TGDSGDGRTGIYTYWFDVFGQGIAHNEKRD-----------FTPTDRQ-------K 105
Psp_EEX53869 EAGDADAFLHARTHFTPYPIPTNKDETQSGNQTGIYTYWFDVFGKGISNNEKRD-----------FTPTARQ-------P 107
Bfi_EEX45726 ---------------------------GKTQT-------VTMEGSD-------------------DES-----------A 49
Bst_EDS13961 ---------------------------GETEIHPDTSEWIYTDGSV-------------------SEPKAKE-------T 64
Pta_EEX71812 ---------------------------GKTETLGVKGPWVYKQQNDEGKMEEA-------YTKEKPENTEVP-------K 83
Por_EFB32073 ---------------------------GDTVTAKDVNEWEYHEFIIENGKYKKKPDGTLDYKVIKTIPAGKP-------N 82
Pti_EFA97464 ---------------------------GKTETQTDYRAWVYGMMNRQTGEMD---------QITKEIPERAN-------N 82
Lbo_ABJ77549 --------------------------------DGSFITKVRSASYDVWIKYNFAN----------KTQAFVP-------D 72
Lbo_ABJ80492 --------------------------------DGSFITKVRSASYDVWIKYNFAN----------KTQAFVP-------D 72
Lin_AAS72180 --------------------------------DGSFTTRIRSVSYDVWIKYNFAD----------KSQAFVP-------D 75

Ssp_EEI93473 --SRWDLSFDDIYR--SFLNCN------TPLRGGTGKGGIL-IVKQKFEDVTDVPAD--------------------NLF 114
Ssp_EER70254 --ARWDLSFDDIYR--SFLNCN------TPLTGGSGKGGIL-IVKQKFDDVTDVPAD--------------------NLF 114
Ssp_EEI91310 --TDWDIAFSGLYN--STLYVNNGQLNGNPASGNTSRHKVI-LVKESYDRVSTAPSD--------------------ADF 125
Ssp_EER72820 --TDWDIAFSGLYN--STLYVNNGQLNGNPAAGNTSRHKVI-LVKESYDRVNTAPSD--------------------ADF 125
Bfr_CAH07859 --TDWDLAFCGYV-----MRTN------SGTSGIGRGGAAD-LGYGNYENWTSVAQLPSDLKWVED-NQEVYVTMSQNDW 122
Bsp_EEZ27613 --TDWDLAFCGYV-----MRTN------SGTSGIGRGGAAD-LGYGNYENWTSVAQLPSDLKWVED-NQEVYVTMSQNDW 122
Bco_EDV01216 --TDWDLAFCGYT-----MRTN------SGTSGIGQGGAAD-LGYGGYDDWTSVSQLPSDLEWVVD-TDDVRVTMSQNDW 119
Pbi_EFB92697 --TDWDIAFCGYR-----MRTN------SGTSGNGKGGAAD-LGYGSYDTWKTVAQLPTNLQWVVD-DHSVYITMSQNDW 121
Por_EFB31294 --LDWDLAFCGYR-----LRTN------SGTSGNGQGGAAD-LGSGNYEKWQTVSQLPNTIQWAVD-DHTVSITYSRNDW 121
Bfr_ZP_05280704 --TDWDLAFCGYR-----LRTN------SGTSGIGQGAAAD-LGYGSYDKWKTVSQLPADLKWTVDNDTEVYVTMSQNDW 122
Pbe_EFA44395 --LDWDIAFCGAH-----IRTN------GGTSGPGKGAAAD-LGFGDYDHWQDRVQIPDDVKWVEDDTTSVYVTYSQREW 122
Pti_EFA97970 --LDWDIAFCGYH-----VRTN------SGTSGKGKGGVID-LGFGDYDHWQRVEQLPKTEKWIVDNDTTVMITYSQNDW 117
Bfr_CAH06766 --TDWDIAFNRYN-----VRTN------SGASGKGKGGALL-TNIKDLAACTTVPQG----------------------- 98
Bfr_BAD47866 --TDWDIAFNRYN-----VRTN------SGASGKGKGGALL-TNIKDLAACTTVPQG----------------------- 98
Bsp_EES87618 --TDWDIAFNRYN-----VRTN------SGASGKGKGGALL-TNIKDLAACTTVPQG----------------------- 98
Bsp_EEZ26547 --TDWDIAFNRYN-----VRTN------SGASGKGKGGALL-TNIKDLAACTTVPQG----------------------- 98
Bfr_ZP_05280033 --TDWDIAFNRYN-----VRTN------SGASGKGKGGALL-TNIKDMAACTTVPQG----------------------- 101
Pue_EEK17166 --TDWDIAIHSFY-----FRAN------CGTSGKGKGGALM-TNQTKLSAVKEAPTE----------------------- 96
Cgi_EEK15165 --LDWDIAFFAYYAKLN-----------GGASGKGQAGVAK-TENKDFSAPIATLPSE------------------YIQD 96
Coc_ACU93343 --LDWDIAFFAHYIKTN-----------GGISGKGEGGAIK-TDSNNFDTVTSAPTEG------------------YTQD 96
Cgi_EEK14790 --LSWDIAFQRWYIKTN-----------SGTSGKGKGGAIN-TKKTDWNKVVYASPSG------------------YKED 95
Csp_EEB66232 --TDWDIAFNRYSVRTN-----------SGTSGSGNGGALT-TNETDWDKVATASSTASFTVDGSI---YVFERGQNNQG 110
Pgi_ABL74281 --LNWDMALHRYDVRLN-----------CGESGKGKGGAVF-SGKTEMDQATTVPTDG------------------YTVD 98
Pgi_CAM31898 --LNWDMALHRYDVRLN-----------CGESGKGKGGAVF-SGKTEMDQATTVPTDG------------------YTVD 98
Pgi_AAQ66587 --LNWDMALHRYDVRLN-----------CGESGKGKGGAVF-SGKTEMDQATTVPTDG------------------YTVD 98
Pgi_CAM31897 --LNWDMALHRHDVRLN-----------CGESGKGKGGAVF-SGKTEMDQATSVPTDG------------------YTVD 98
Pgi_BAG33077 --LNWDMALHRYDVRLN-----------CGESGKGKGGAVF-SGKTEMDQATSVPTDG------------------YTVD 98
Pen_EEN82265 --DAWDIAFHRFDIRLN-----------CGESGKGKGAAVF-SGVTEMEKATKVPTEG------------------WVTD 112
Pue_EEK17583 --LSWDLGLHRYDFKTN-----------GGDSGKGKGAAVRITKQKVLTADIPTPEDSE-----------------WTLD 110
Pue_EEK16193 --LSWDLGLHRYDFKTN-----------GGTSGKGKGAAAR-TSQKDLMADIPTPKDSE-----------------WALD 102
Bsp_EEO56990 IPDKWDFAIHRY-------------------DIKTNEGAAYKTTYTSIDEFKATG---------------------KLPK 97
Bsp_ZP_0561712 IPDKWDFAIHRY-------------------DIKTNEGAAYKTTYTSIDEFKATG---------------------KLPK 97
Bov_EDO10376 IPDKWDFAIHRY-------------------DIKTNEGAAYKTTYTSIDEFKATG---------------------KLPK 97
Bca_EDM22026 IPDNWDIAIHRY-------------------DIKTNEGAAYQTSYTSFDALKANG---------------------KLPD 97
Bsp_EES70848 IPAKWDLAIHRY-------------------DIKTNEGAAFQTTYTSIDDLKASG---------------------KLPA 97
Bth_AAO75604 IPAKWDLAIHRY-------------------DIKTNEGAAFQTTYTSIDDLKASG---------------------KLPA 97
Bdo_EEB24050 -PEEWDIAVHRY-------------------DVKTNAGAVLETGFTGFSALQNAS---------------------AMPE 105
Bsp_EEZ23549 -PEEWDIAVHRY-------------------DVKTNAGAVLETGFTGFSALQNAS---------------------AMPE 105
Bsp_EEO61578 -PEEWDIAVHRY-------------------DVKTNAGAVLETGFTGFSALQNAS---------------------AMPE 105
Bsp_EEO47827 -PEEWDIAVHRY-------------------DVKTNAGAVLETGFTGFSALQNAS---------------------AMPE 105
Bsp_EET16222 -PEEWDIAVHRY-------------------DVKTNAGAVLETGFTGFSALRNAD---------------------AMPE 105
Bvu_ABR39853 -PEEWDIAVHRY-------------------DVKTNAGAVLETGFTGFSALRNAD---------------------AMPE 105
Bsp_EEZ28283 -PDTWDLAIHRY-------------------DVKTNGGEVLETDYQSLSALKNAG---------------------SMPQ 101
Bsp_EES84662 -PDTWDLAIHRY-------------------DVKTNGGEVLETDYQSLSALKNAG---------------------SMPQ 101
Bfr_CAH08403 -PDTWDLAIHRY-------------------DVKTNGGEVLETDYQSLSALKNAG---------------------SMPQ 101
Bfr_BAD49437 -PDTWDLAIHRY-------------------DVKTNGGEVLETDYQSLSALKNAD---------------------SMPQ 101
Bfr_ZP_05281773 -PAAWDLAIHRY-------------------DVKTNGGEVLETDFQSLNALKNAD---------------------SMPQ 103
Bcp_EEF75672 LPQEWDFAIHRY-------------------DTKTNEGAVLETSAESMEELLAGK---------------------EIPQ 146
Por_EFB31210 IPAEWHFALHRY-------------------DCKTNGGAALETAYADLETFRRDAGNGTY----------------ARPS 109
Pme_EES82771 EPQSWSIAFHRN-------------------NVRTNGGAVLETKYTSLNELPKNS---------------------SYFL 148
Pve_EEX17867 KPPSWSLAFHRN-------------------NVRTNGGAVLETNYKSMSELPKSS---------------------IDFL 147
Pta_EEX72705 EPLSWTFAVHRN-------------------NVRTNGGAVLETNYNDLSELPANS---------------------ADFL 145
Psp_EEX53869 EPPAWSIAIHRD-------------------NVRTNGGAVLETNYTSMSQLPQSS---------------------AQFM 147
Bfi_EEX45726 VTIDWQIAIHRYT------------------EVKTNGGSVVKT---DMTDMSKVT---------------------TIPT 87
Bst_EDS13961 IGIEWHIAIHRY-------------------EIKTNGGMVFDT---EKTNMNEIT---------------------ELPE 101
Pta_EEX71812 ELKDWQLAFHRF-------------------EPKTNMGEVVET---TETELDKVT---------------------TIPS 120
Por_EFB32073 APKTWHLAFHVY-------------------DALINNGEALMT---NETELSKLT---------------------EMPK 119
Pti_EFA97464 EPKKWHIAFHLY-------------------DPMTNGGEVMIAGK-DTTSLDQIT---------------------ELPK 121
Lbo_ABJ77549 TSGGWDVGFQRF-------------------KLQTNGGLTYSEGQGGACLTNPVLT--------------------DFNV 113
Lbo_ABJ80492 TSGGWDVGFQRF-------------------KLQTNGGLTYSEGQGGACLTNPVLT--------------------DFNV 113
Lin_AAS72180 MSGGWDVGFQRF-------------------KLQTNSGLTHSEGQGGACMTNPVVT--------------------DFET 116

Ssp_EEI93473 RTGEKSYGTDDSG-----------------AFG--EGLGWYLYDFGGTIKGGSDPRKAHVCYPIEGHTIIVRTANG-NYA 174
Ssp_EER70254 RTGEKSYGTDDSG-----------------AFG--EGLGWYLYDFGGTIKGGSDPRKAHVCYPIEGHTIIVRTAKG-NYA 174
Ssp_EEI91310 DKSQ----LNDFG-----------------MIINEDSQGWYDYNVTN-----------HLVAVVPNRTYVIRLTNG-KYA 172
Ssp_EER72820 DKSQ----LNDFG-----------------MIGNEDSQGWYNYNLTN-----------HLVAVVPNRTYVIRLTNG-KYA 172
Bfr_CAH07859 NHYLIENGLDFN------------SN---PWFDPNNGPQKTTTNANPVLAQAMSFAGPPPVYTPSYHTYVVRTADGKHYF 187
Bsp_EEZ27613 NHYLIENGLDFN------------SN---PWFDPNNGPQKTTTNANPVLAQAMSFAGPPPVYTPSYHTYVVRTADGKHYF 187
Bco_EDV01216 NHYLVENNLDFD------------AN---PWFDPNNGPATTETNANPLLSQAMSFSGPPPTYTPSFHTYVVRTADGERYF 184
Pbi_EFB92697 NKYLIANKLDFK------------QN---PWFDPNRGPASTQTDANPTLAKAMTFTGPPPVYAPSFHTYVVRTADGQRYF 186
Por_EFB31294 NKYLIANHLDFN------------EN---PWFDPNRGPATTKTDANPVLARAMTFTGPPPVYSPSFHTYVVRTADGKRYF 186
Bfr_ZP_05280704 NKYLIEHGLDFN------------EN---PWFDPNTGPAKTLTSANPILAEAMSFAGPPPVYTPSYHTYVIRTADGKRYF 187
Pbe_EFA44395 YNYVNTHYLKPDGT---------PDNDGHPWFDPNRGPARRLTSGNPLLEQCIKIEGPPMTYTPSYHVYVIRCADGVRCF 193
Pti_EFA97970 FRYVNTHKLDPK------------EN---PWFDPNNGPQRTLTSANPLLERNMFLSGPPMTYTPSYHVYVVRTADGKRYF 182
Bfr_CAH06766 -TFTVDAAYTIT------------------APGTGFPPPTMESTANEVLCKAITFAGPPPTYTPSDYVFIVRTASG-KYA 158
Bfr_BAD47866 -TFTVDAAYTIT------------------APGTGFPPPTMESTANEVLCKAITFAGPPPTYTPSDYVFIVRTASG-KYA 158
Bsp_EES87618 -TFTVDAAYTIT------------------APGTGFPPPTMESTANEVLCKAITFAGPPPTYTPSDYVFIVRTASG-KYA 158
Bsp_EEZ26547 -TFTVDAACTIT------------------APGTGFPPPTMESTANEVLCKAITFAGPPPTYTPSDYVFIVRTASG-KYA 158
Bfr_ZP_05280033 -TFTVDASYTIT------------------APGTGFPPPTMESTANEVLCKAITFAGPPPTYTPSDYVFIVRTASG-KYA 161
Pue_EEK17166 -GYIVDEAISIW------------------GWKG--ELIKAEVSGNPELNKMIGFSGPPPKYTPSDNIFIIRTADG-KYA 154
Cgi_EEK15165 VKGTMSYG---------NYPNLTEKEDTFSTFLSGGFDTPTGYVSLNPNNRQSSGGKWPSVYAPTKWVYVLKTAKG-AFV 166
Coc_ACU93343 VKGTMSYG---------SYPNLTKKEGTFSPIVSGDFETKTGYVSLSPNN----IGKWPSVYAPTKYVYIIKTAKG-EYA 162
Cgi_EEK14790 AIGTLNG------------WDIIK---NVETKKEGSFSQ-EASLYVTYIS---GGK-----YKNRNEVYLLKTAEG-KFV 150
Csp_EEB66232 GLGTTNASRVISGAHGENFWNMISRMPNVAKIDKSSIVHNNGWLTMDYKP---NGNQLAPSYTYNNWVYIVKTPAG-KFV 186
Pgi_ABL74281 VLGRITVKYEMG-----PDGHQMEYEEQGFSEVITGKKNAQGFASGGWLEFS--HGPAGPTYKLSKRVFFVRGADG-NIA 170
Pgi_CAM31898 VLGRITVKYEMG-----PDGHQMEYEEQGFSEVITGKKNAQGFASGGWLEFS--HGPAGPTYKLSKRVFFVRGADG-NIA 170
Pgi_AAQ66587 VLGRITVKYEMG-----PDGHQMEYEEQGFSEVITGKKNAQGFASGGWLEFS--HGPAGPTYKLSKRVFFVRGADG-NIA 170
Pgi_CAM31897 VLGRITVKYEMG-----PDGHQMEYEEQGFSEVITGKKNAQGFASGGWLEFS--HGPAGPTYKLSKRVFFVRGADG-NIA 170
Pgi_BAG33077 VLGRITVKYEMG-----PDGHQMEYEEQGFSEVITGKKNAQGFASGGWLEFS--HGPAGPTYKLSKRVFFVRGADG-NIA 170
Pen_EEN82265 AIGTITIKFSMG-----GGSHDSNHEQTGYNHLITGKKTERGDMSGGWLDYD--LGNMPPRVRLSGKVFFVKCADG-KIA 184
Pue_EEK17583 REGLLLMQFEDDG----LGSHRTKYEMQEANFLLSSECDGYG-GYTNKGVIW--QEGMPPQVYWDNGIYLVRSASG-EIA 182
Pue_EEK16193 REGTLLMKFD-------MSKHEMKYEKQSANFLLTSEPKDDGKGYLNKGIIS--RAGMPPTVTVDPSVFLVRSATG-QIV 173
Bsp_EEO56990 AEDFVEDE-WTTDKIAIDMSGMMDGN--IIYTESYRNAVLS-------SWLDVNTATMPPVYTMSNQVFLIRLKDN-TYA 166
Bsp_ZP_0561712 AEDFVEDE-WTTDKIAIDMSGMMDGN--IIYTESYRNAVLS-------SWLDVNTATMPPVYTMSNQVFLIRLKDN-TYA 166
Bov_EDO10376 AEDFVKDE-WTTDKIAIDMSGMMDGN--IIYTESYRNAVLS-------SWLDVNTATMPPVYTMSNQVFLIRLKDN-TYA 166
Bca_EDM22026 DKDFVKDE-WTTDKIAIDMSGMMEGN--IVYTDSYYNSVLS-------TWLNVDTSTMPPIYTMSNQVYLIRLKDN-TYA 166
Bsp_EES70848 EENFVKDE-WTTDKIAIDMSGMMEGN--IKYTEDYRNDILS-------GWLNVDTSSMPPIYTMSNQVYLIQLKDN-TYA 166
Bth_AAO75604 EENFVKDE-WTTDKIAIDMSGMMEGN--IKYTEDYRNDILS-------GWLNVDTSSMPPIYTMSNQVYLIQLKDN-TYA 166
Bdo_EEB24050 G-VYVDDV-WTTAKVAIDMSGMMDGN--IVYMESYYNEELS-------KWLNVDKSNMPPTYTLSNKVYMVKLKDG-TYA 173
Bsp_EEZ23549 G-VYVDDV-WTTAKVAIDMSGMMDGN--IVYMESYYNEELS-------KWLNVDKSNMPPTYTLSNKVYMVKLKDG-TYA 173
Bsp_EEO61578 G-VYVDDV-WTTAKVAIDMSGMMDGN--IVYMESYYNEELS-------KWLNVDKSNMPPTYTLSNKVYMVKLKDG-TYA 173
Bsp_EEO47827 G-VYVDDV-WTTAKVAIDMSGMMDGN--IVYMESYYNEELS-------KWLNVDKSNMPPTYTLSNKVYMVKLKDG-TYA 173
Bsp_EET16222 G-AYVEDV-WTTAKIAIDMSGMMDGN--IVYMASYYNEELS-------KWLNVDKSNMPPTYTLSNKVYMVKLKDG-TYA 173
Bvu_ABR39853 G-AYVEDV-WTTAKIAIDMSGMMDGN--IVYMESYYNEELS-------KWLNVDKSNMPPTYTLSNKVYMVKLKDG-TYA 173
Bsp_EEZ28283 G-TFVADE-WTTNKIAVDVSHMMEDNGYLIYAPSDFNPELS-------KWLNVDTSEMPPIYTPSNKVYLLRMKDG-TMA 171
Bsp_EES84662 G-TFVADE-WTTNKIAVDVSHMMEDNGYLIYAPSDFNPELS-------KWLNVDTSEMPPIYTPSNKVYLLRMKDG-TMA 171
Bfr_CAH08403 G-IFVADE-WTTNKIAVDVSHMMEDNGYLIYAPSDFNPELS-------KWLNVDTSEMPPIYTPSNKVYLLRMKDD-TMA 171
Bfr_BAD49437 G-TFVADE-WTTNKIAVDVSHMMEDNGYLIYAPSDFNPELS-------KWLNVDTSEMPPIYTPSNKVYLLRMKDG-TMA 171
Bfr_ZP_05281773 G-TFVADE-WTTNKIAIDVSHMMEDDGYLIYAPSDYNPELS-------KWLDVDTREMPPIYTPSNKVYLLRMEDG-TMA 173
Bcp_EEF75672 G-NYTADTPGTTDKITIDLSDMYNGT--IRYACSDYNSVLS-------QWLSR--KGMPPTYTLSGKVYVLRLKDG-THA 213
Por_EFB31210 DASFKPDV---ADRIIIDMSGMMQGK--VIYANTPKNKELG-------KWLDVNMSTMPPTYKPSEKVYLLLMKDK-TIA 176
Pme_EES82771 GATFQEDE-WTENEVWEDQSQMLMSL--IGCQGIRINKVLS-------SWLKIEIPPMPPSFTMNSHVFILRLNNG-KYA 217
Pve_EEX17867 GANFQTDE-WSENEVWFDQSQMLLSL--IGCQGITINKVLS-------SWLRLDIPPMPPAFTMKSNVYILRLKNG-KYA 216
Pta_EEX72705 GAAFKEDE-WTENTVWVDQSKMLNEL--IGCQGIKTNTVLS-------SWLRLEIPPMPPAFIHNKHVFILRLKSG-LYA 214
Psp_EEX53869 GADFKSDE-WTQNEVWVDQSQMLNSL--IGCQGISINRPLS-------SWLRLEIPPMPPAFKHNNHVFIVKLNNG-KFV 216
Bfi_EEX45726 GGYVADES---EQNILVRFAMPPTDDCYVKS---HCNTAMV---------WTEGSAMAGTLAT-TNNVFVLKCKDG-TYA 150
Bst_EDS13961 GDYKADEN---ITNEDEEYAIITDMSKMMQGNVGYAKTATVNKVLCSWVKKTETGSMPPTIYEPTMHVIVLKCKDG-SWA 177
Pta_EEX71812 SGFEADKD---IKEQ-----IIVDAKNMMMGHIGYAKIAKVNLALYKWLKRTPTGGMPPYEYSIPGKVFILKCKDG-QYY 191
Por_EFB32073 G-NYVANK---QLFIPVDMSGMQNG--VMGYSKGFSNLELN-------KWISSKGMPPKYTMSDK--VFSVRFKDG-SYA 183
Pti_EFA97464 GGTWTADK---PVWILVDMAGMMQMPPTMGYSKGFSNPNLH-------KYMRRAGMG-QYEMANKGRIFIVKFKDG-SFA 189
Lbo_ABJ77549 AASSTSRALGCTNAFFSPDTNVSEIAAGGIQTNYVGNNVLN-------KWFNYTFAFLQPNYN----IFIVRSNTGNEYY 182
Lbo_ABJ80492 AASSTSRALGCTNAFFSPDTNVSEIAAGGIQTNYVGNNVLN-------KWFNYTFAFLQPNYN----IFIVRSNTGNEYY 182
Lin_AAS72180 AASSTSTALGCVNASFSPDTNVSELASGGIQTNYVGNDVLN-------KWFNYSLAFLQPNYK----VFVIRSNTGNEYY 185

Ssp_EEI93473 KVRIQSIYKDLLDPKDWYKDSPTPYFTFQYVLVKAGSKTFEIKK--- 218
Ssp_EER70254 KVRIQSIYKDLLDPKDWYKDSPTPYFTFQYVLVKAGSKTFEIKK--- 218
Ssp_EEI91310 KLQIVNIYKGNP-PAVTDLNWPAPYFTFKYFVQEDGSKNLKTK---- 210
Ssp_EER72820 KLQIVNIYKGNP-PAVTDLNWPAPYFTFKYFVQEDGSKNLKTK---- 214
Bfr_CAH07859 KIQIISWYDANVEIGDEGG-------RLSYYCDELQP---------- 217
Bsp_EEZ27613 KIQIISWYNANVEIGDEGG-------RLSYYCDELQP---------- 217
Bco_EDV01216 KIQIISWYDANVEIGDEGG-------RISYYCDELK----------- 213
Pbi_EFB92697 KLQIISWYKADVEIGDTGG-------QMSYYCDELK----------- 215
Por_EFB31294 KLQIISWYKGDIEVGDTGG-------QISYYCDELK----------- 215
Bfr_ZP_05280704 KIQIISWYNADSEIGDSGG-------QISYYCDELN----------- 216
Pbe_EFA44395 KLQVVSWFNQQSSIDDEGGG------QMSYYIDELK----------- 223
Pti_EFA97970 KLQIVSWYNQHTEIDDTGG-------QISYYCDELK----------- 211
Bfr_CAH06766 KLKAKSFYDDE---GKSGIY------SFEYAIQPDGSTNLN------ 190
Bfr_BAD47866 KLKAKSFYDDE---GKSGIY------SFEYAIQPDGSTNLN------ 190
Bsp_EES87618 KLKAKSFYDDE---GKSGIY------SFEYAIQPDGSTNLN------ 190
Bsp_EEZ26547 KLKAKSFYDDE---GKSGIY------SFEYAIQPDGSTNLN------ 190
Bfr_ZP_05280033 KLKAKSFYDDE---GNSGIY------SFEYAIQPNGSTNLN------ 193
Pue_EEK17166 KVKMISYIDDS---GKSGIV------SFDYVYQPDGSTKLD------ 186
Cgi_EEK15165 KFQVTDFYNDK-----TDA----NYPSFQYILSEDGKF--------- 195
Coc_ACU93343 KFQVTDFYNDG-----AKP----NYVTFSYLISKDGKF--------- 191
Cgi_EEK14790 KIQFYDYVNER-----LKG----GYPSFRYKISNDEN-F-------- 179
Csp_EEB66232 KIQLTDYKNAK-----DET----GYITFKYQIANDKNEFK------- 217
Pgi_ABL74281 KVQFTDYQDAE-----LKK----GVITFTYTYPVK------------ 196
Pgi_CAM31898 KVQFTDYQDAE-----LKK----GVITFTYTYPVK------------ 196
Pgi_AAQ66587 KVQFTDYQDAE-----LKK----GVITFTYTYPVK------------ 196
Pgi_CAM31897 KVQFTDYQDAE-----LKK----GVITFTYTYPVK------------ 196
Pgi_BAG33077 KVQFTDYQDAE-----LKK----GVITFTYTYPVK------------ 196
Pen_EEN82265 RIQFTDCKDKT-----GRKN---GFISFTYDYNVSVK---------- 213
Pue_EEK17583 RLRVLDYQDNK-----KHR----GYITIEYAIQVDKK---------- 210
Pue_EEK16193 RVRVLDYQNAT-----KKT----GYITLEYIIKADKK---------- 200
Bsp_EEO56990 AIRFTNYMN------ARGIK---GYIDFNFQYPLEFEDNNNETNQQE 204
Bsp_ZP_0561712 AIRFTNYMN------ARGLK---GYIDFDFQYPLEFEDNNNETNQQE 204
Bov_EDO10376 AIRFTNYMN------ARGIK---GYIDFDFQYPLEFEDNNNETNQQE 204
Bca_EDM22026 AIRFTNYTN------AKGIK---GYIDFDFLYPFDFEENN------- 197
Bsp_EES70848 AIRFTNYTN------ARGIK---GYIDFDFLYPLDFEENN------- 197
Bth_AAO75604 AIRFTNYTN------ARGIK---GYIDFDFLYPLDFEENN------- 197
Bdo_EEB24050 AVRLTNYMN------ASGVK---GFMTIDYIYPFEL----------- 200
Bsp_EEZ23549 AVRLTNYMN------ASGVK---GFMTIDYIYPFEL----------- 200
Bsp_EEO61578 AVRLTNYMN------ASGVK---GFMTIDYIYPFEL----------- 200
Bsp_EEO47827 AVRLTNYMN------ASGVK---GFMTIDYIYPFEL----------- 200
Bsp_EET16222 AVRLTNYMN------ASGVK---GFMTIDYIYPFEL----------- 200
Bvu_ABR39853 AVRLTNYMN------ASGVK---GFMTIDYIYPFEL----------- 200
Bsp_EEZ28283 AIRLVSYMN------AAGIK---GYMTFDYIYPYEP----------- 198
Bsp_EES84662 AIRLVSYMN------AAGIK---GYMTFDYIYPYEP----------- 198
Bfr_CAH08403 AIRLVSYMN------AAGIK---GYMTFDYIYPYEP----------- 198
Bfr_BAD49437 AIRLVSYMN------AAGIK---GYMTFDYIYPYEP----------- 198
Bfr_ZP_05281773 AIRLTSYMN------AKGAK---GYMTFDYLYPYEP----------- 200
Bcp_EEF75672 ALKLTDYRN------EMYVN---GYMRVQYIYPLENALK-------- 211
Por_EFB31210 AIRFTGFANPN----KYNIK---GYISFDYIYPVKFKD--------- 207
Pme_EES82771 ALQLENYIG------TDGTK---CWLRINYKYPY------------- 242
Pve_EEX17867 ALQLVNYMN------TGGTK---CWLTINYKYPY------------- 241
Pta_EEX72705 ALQLENYIG------ADGKK---CNLTIRYKYPY------------- 239
Psp_EEX53869 ALQLESYINP-----TNGTK---CHLTINYRYPY------------- 242
Bfi_EEX45726 KLQFTDFAN------ADNVK---GHVTFNYEY-PVK----------- 176
Bst_EDS13961 KLQFTVAGN------SETNKS--GFVTFNYEFIPIK----------- 205
Pta_EEX71812 KLKFTDFMD------KEGKK---GVITFSYVPIKAKK---------- 219
Por_EFB32073 LIKFKDYAD------KKGKK---KLVDFEYKFVKK------------ 209
Pti_EFA97464 AIKFTDITD------ATGKK---KQVSFDYKFVKKN----------- 216
Lbo_ABJ77549 LFQITGYYN------SEGSS---AHPTVRWKQIPY------------ 208
Lbo_ABJ80492 LFQITGYYN------SEGSS---AHPTVRWKQIPY------------ 208
Lin_AAS72180 LFQITGYYN------SEGTS---AHPTIRWKQIQY------------ 211

**Fig. 1A**

Cgi_EEK15165 -----------CSKDSN--------KEEEKKPANVKQEKNLNAR-QEKWVYYSFEKN----------------------- 37
Coc_ACU93343 -----------CSKDND--------SKEEKKISKGTQVKNLYTFNKDNWVYFSFKK------------------------ 37
Cgi_EEK14790 -----------CEKE----------KGEETILSVTKDVKMLNATSYQKWVYYSFEKG----------------------- 36
Csp_EEB66232 -----------CSKD----------DKKTEAPKLPYKEQKVDASSYDKWVYFSFENG----------------------- 36
Pme_EES82771 -----------CNGIFEDIY-DEAPATANVTT---EGQLLVNAASWKDWYYVDFDSLQMYIERKDTAGLLKAQTNFTHYA 65
Pve_EEX17867 -----------CNGLFDGIY-DDAPASPTIT----EGQLLVDATSWKDWYYVDFDSLQAYIEKKDTVGLLKAQSHFTPYH 64
Psp_EEX53869 -----------CTGLFDGIY-DSPDKAPKIN----ANQLAIDASDWHNWYYIDFDSLQMLAEAGDADAFLHARTHFTPYP 64
Pta_EEX72705 -----------CNGILGGIY-DEQPDKAAVA----PGTLYIDASGWTDWHYVDLDSLAALQAAGDSVGLRRAQTHFTHFP 64
Por_EFB32073 -----------CSKDDNTP--VNPQN-------VNSKRLTLNASPRDSWLYVNLETGDTVTAK------DVNEWEYHEFI 54
Pta_EEX71812 -----------CSKDDPAPK-PNPKPDPNPEVPAQVKQITVTATEYDTWTYVDLKTGKTETLG------VKGPWVYKQQN 62
Pgi_ABL74281 -----------CGKKKD-------EPNQPSTPEAVTKTVTIDASKYETWQYFSFS------------------------- 37
Pgi_CAM31898 -----------CGKKKD-------EPNQPSTPEAVTKTVTIDASKYETWQYFSFS------------------------- 37
Pgi_AAQ66587 -----------CGKKKD-------EPNQPSTPEAVTKTVTIDASKYETWQYFSFS------------------------- 37
Pgi_CAM31897 -----------CGKKKD-------EPNQPSTPEAVTKTVTIDASKYETWQYFSFS------------------------- 37
Pgi_BAG33077 -----------CGKKKD-------EPNQPSTPEAVTKTVTIDASKYETWQYFSFS------------------------- 37
Pen_EEN82265 ----MVLGVASCRPNQTPKPGPTPTPNPPAQEEVKGLKGYVDASKYEKWVYYSLK------------------------- 51
Por_EFB31210 MTGTIVLSFLSCDNIYDDPS-----EGDPSQHKKDNTYTNINATEYTNWVYLNLKNG----------------------- 52
Por_EFB31294 -----------CVKY----------DAEPFTGKTLPRVSGYSTGVTNDWIYFNLRTGEVFN------------------- 40


Cgi_EEK15165 ----------------------------------AIVEVADPQNSLDWDIAFFAYYAKLNGGASGKGQAGVAKTENKDFS 83
Coc_ACU93343 ----------------------------------GVITIQDPENSLDWDIAFFAHYIKTNGGISGKGEGGAIKTDSNNFD 83
Cgi_EEK14790 ----------------------------------AVVEVSSPETDLSWDIAFQRWYIKTNSGTSGKGKGGAINTKKTDWN 82
Csp_EEB66232 ----------------------------------VVTSTTATPTTTDWDIAFNRYSVRTNSGTSGSGNGGALTTNETDWD 82
Pme_EES82771 IPTNLTSGSGDGKTGMYTYWFDVFGKGISVNEKRGFTSTDAQPEPQSWSIAFHRNNVRTNGGA-------VLETKYTSLN 138
Pve_EEX17867 IPSSLSTGTSNGQTGIYTYWFDVFGKGIAVNEKRSFKVTDAQPKPPSWSLAFHRNNVRTNGGA-------VLETNYKSMS 137
Psp_EEX53869 IPTNKDETQSGNQTGIYTYWFDVFGKGISNNEKRDFTPTARQPEPPAWSIAIHRDNVRTNGGA-------VLETNYTSMS 137
Pta_EEX72705 IPTTGDSG--DGRTGIYTYWFDVFGQGIAHNEKRDFTPTDRQKEPLSWTFAVHRNNVRTNGGA-------VLETNYNDLS 135
Por_EFB32073 IENGKYKKKPDGTL---------------DYKVIKTIPAGKPNAPKTWHLAFHVYDALINNGE-------ALMTNETELS 112
Pta_EEX71812 DE-GKMEEA---------------------YTKEKPENTEVPKELKDWQLAFHRFEPKTNMGE-------VVETTETELD 113
Pgi_ABL74281 --------------------------------KGEVVNVTDYKNDLNWDMALHRYDVRLNCGESGKGKGGAVFSGKTEMD 85
Pgi_CAM31898 --------------------------------KGEVVNVTDYKNDLNWDMALHRYDVRLNCGESGKGKGGAVFSGKTEMD 85
Pgi_AAQ66587 --------------------------------KGEVVNVTDYKNDLNWDMALHRYDVRLNCGESGKGKGGAVFSGKTEMD 85
Pgi_CAM31897 --------------------------------KGEVVNVTDYKNDLNWDMALHRHDVRLNCGESGKGKGGAVFSGKTEMD 85
Pgi_BAG33077 --------------------------------KGEVVNVTDYKNDLNWDMALHRYDVRLNCGESGKGKGGAVFSGKTEMD 85
Pen_EEN82265 --------------------------------DNKEVEVSDYKNSDAWDIAFHRFDIRLNCGESGKGKGAAVFSGVTEME 99
Por_EFB31210 -------------------------------SQKTLLYDNTADIPAEWHFALHRYDCKTNGGAALETAYADLETFRRDAG 101
Por_EFB31294 -----------------------------SYQPGSDIKEGEQRNRLDWDLAFCGYRLRTNSGTSGNGQGGAADLGSGNYE 91


Cgi_EEK15165 APIATLPSE---------------YIQDVKGTMSYG---------NYPNLTEKEDTFSTFLSGGFDTPTGYVSLNPNNRQ 139
Coc_ACU93343 TVTSAPTEG---------------YTQDVKGTMSYG---------SYPNLTKKEGTFSPIVSGDFETKTGYVSLSPNN-- 137
Cgi_EEK14790 KVVYASPSG---------------YKEDAIGTLNG------------WDIIK---NVETKKEGSFSQ-EASLYVTYIS-- 129
Csp_EEB66232 KVATASSTASFTVDGSIYVFERGQNNQGGLGTTNASRVISGAHGENFWNMISRMPNVAKIDKSSIVHNNGWLTMDYKP-- 160
Pme_EES82771 ELPKNSSYF---------------LGATFQEDEWTE-----------NEVWEDQSQMLMSLIGCQG-IRINKVLSSWLK- 190
Pve_EEX17867 ELPKSSIDF---------------LGANFQTDEWSE-----------NEVWFDQSQMLLSLIGCQG-ITINKVLSSWLR- 189
Psp_EEX53869 QLPQSSAQF---------------MGADFKSDEWTQ-----------NEVWVDQSQMLNSLIGCQG-ISINRPLSSWLR- 189
Pta_EEX72705 ELPANSADF---------------LGAAFKEDEWTE-----------NTVWVDQSKMLNELIGCQG-IKTNTVLSSWLR- 187
Por_EFB32073 KLTEMP-------------------KGNYVANKQL-------------FIPVDMSGMQNGVMGYSK-GFSNLELNKWIS- 158
Pta_EEX71812 KVTTIP-------------------SSGFEADKDIK-----------EQIIVDAKNMMMGHIGYAKIAKVNLALYKWLKR 163
Pgi_ABL74281 QATTVPTDG---------------YTVDVLGRITVK-----------YEMGPDGHQMEYEEQGFSEVITGKKNAQGFASG 139
Pgi_CAM31898 QATTVPTDG---------------YTVDVLGRITVK-----------YEMGPDGHQMEYEEQGFSEVITGKKNAQGFASG 139
Pgi_AAQ66587 QATTVPTDG---------------YTVDVLGRITVK-----------YEMGPDGHQMEYEEQGFSEVITGKKNAQGFASG 139
Pgi_CAM31897 QATSVPTDG---------------YTVDVLGRITVK-----------YEMGPDGHQMEYEEQGFSEVITGKKNAQGFASG 139
Pgi_BAG33077 QATSVPTDG---------------YTVDVLGRITVK-----------YEMGPDGHQMEYEEQGFSEVITGKKNAQGFASG 139
Pen_EEN82265 KATKVPTEG---------------WVTDAIGTITIK-----------FSMGGGSHDSNHEQTGYNHLITGKKTERGDMSG 153
Por_EFB31210 NGTYARPSD-----------------ASFKPDVADR-------------IIIDMSGMMQGKVIYAN-TPKNKELGKWLDV 150
Por_EFB31294 KWQTVSQLPN----------TIQWAVDDHTVSITYSR--------NDWNKYLIANHLDFNENPWFDPNRGPATTKTDANP 153


Cgi_EEK15165 -----SSGGKWPSVYAPTKWVYVLKTAKGA-FVKFQVTDFYN--DKTDANYPSFQYILSEDGKF-- 195
Coc_ACU93343 -------IGKWPSVYAPTKYVYIIKTAKGE-YAKFQVTDFYN--DGAKPNYVTFSYLISKDGKF-- 191
Cgi_EEK14790 ------GGK-----YKNRNEVYLLKTAEGK-FVKIQFYDYVN--ERLKGGYPSFRYKISNDEN-F- 179
Csp_EEB66232 ------NGNQLAPSYTYNNWVYIVKTPAGK-FVKIQLTDYKN--AKDETGYITFKYQIANDKNEFK 217
Pme_EES82771 ----IEIPPMPP-SFTMNSHVFILRLNNGK-YAALQLENYIG--TDGTKCWLRINYKYPY------ 242
Pve_EEX17867 ----LDIPPMPP-AFTMKSNVYILRLKNGK-YAALQLVNYMN--TGGTKCWLTINYKYPY------ 241
Psp_EEX53869 ----LEIPPMPP-AFKHNNHVFIVKLNNGK-FVALQLESYINP-TNGTKCHLTINYRYPY------ 242
Pta_EEX72705 ----LEIPPMPP-AFIHNKHVFILRLKSGL-YAALQLENYIG--ADGKKCNLTIRYKYPY------ 239
Por_EFB32073 ------SKGMPP-KYTMSDKVFSVRFKDGS-YALIKFKDYAD--KKGKKKLVDFEYKFVKK----- 209
Pta_EEX71812 ----TPTGGMPPYEYSIPGKVFILKCKDGQ-YYKLKFTDFMD--KEGKKGVITFSYVPIKAKK--- 219
Pgi_ABL74281 GWLEFSHGPAGP-TYKLSKRVFFVRGADGN-IAKVQFTDYQDA-ELKK-GVITFTYTYPVK----- 196
Pgi_CAM31898 GWLEFSHGPAGP-TYKLSKRVFFVRGADGN-IAKVQFTDYQDA-ELKK-GVITFTYTYPVK----- 196
Pgi_AAQ66587 GWLEFSHGPAGP-TYKLSKRVFFVRGADGN-IAKVQFTDYQDA-ELKK-GVITFTYTYPVK----- 196
Pgi_CAM31897 GWLEFSHGPAGP-TYKLSKRVFFVRGADGN-IAKVQFTDYQDA-ELKK-GVITFTYTYPVK----- 196
Pgi_BAG33077 GWLEFSHGPAGP-TYKLSKRVFFVRGADGN-IAKVQFTDYQDA-ELKK-GVITFTYTYPVK----- 196
Pen_EEN82265 GWLDYDLGNMPP-RVRLSGKVFFVKCADGK-IARIQFTDCKDK-TGRKNGFISFTYDYNVSVK--- 213
Por_EFB31210 N-----MSTMPP-TYKPSEKVYLLLMKDKT-IAAIRFTGFANPNKYNIKGYISFDYIYPVKFKD-- 207
Por_EFB31294 VLARAMTFTGPPPVYSPSFHTYVVRTADGKRYFKLQIISWYKGDIEVGDTGGQISYYCDELK---- 215

**Fig. 1B**

**Fig. 1C**

**Additional file 1: Comparison of HmuY homologues.** Comparison of homologous HmuY amino-acid sequences identified in human pathogens (A) and bacteria identified in oral tissues (B). Amino-acid sequences lacking signal peptides are shown. Positions with identical amino acids in more than 30% of the sequences are shown in black boxes and partial homology is indicated in grey boxes. Phylogenetic relationship between homologous HmuY amino-acid sequences (C). Bacteria infecting the oral cavity are shown in bold. The phylogenetic tree was determined with the Neighbor-Joining method. Bootstrap values are included. Pgi, *Porphyromonas gingivalis*; Pen, *P. endodontalis*; Pue, *P. uenonis*; Bfr, *Bacteroides fragilis*; Bfi, *B. finegoldii*; Bco, *B. coprocola*; Bst, *B. stercoris*; Bdo, *B. dorei*; Bvu, *B. vulgatus*; Bov, *B. ovatus*; Bca, *B. caccae*; Bth, *B. thetaiotaomicron*; Bcp, *B. coprophilus*; Bsp, *Bacteroides* sp.; Coc, *Capnocytophaga ochracea*; Cgi, *C. gingivalis*; Csp, *C. sputigena*; Lbo, *Leptospira borgpetersenii*; Lin, *L. interrogans*; Ssp, *Sphingobacterium spiritivorum*; Pbi, *Prevotella bivia*; Por, *P. oris*; Pbe, *P. bergensis*; Pti, *P. timonensis*; Pme, *P. melaninogenica*; Pve, *P. veroralis*; Psp, *Prevotella* sp.; Pta, *P. tannerae.*
