# Supplementary material for: Species specificity, surface exposure, protein expression, immunogenicity, and participation in biofilm formation of Porphyromonas gingivalis HmuY
Source: BMC Microbiol. 2010 May 4;10:134. doi: 10.1186/1471-2180-10-134 (PMC2873494; doi:10.1186/1471-2180-10-134)
Supplement: Additional file 2 — Analysis of surface exposure of HmuY. Analysis of surface exposure of P. gingivalis HmuY analyzed by whole-cell ELISA. P. gingivalis wild-type (A7436, W83) and hmuY deletion mutant (TO4) strains were grown in basal medium supplemented with hemin (Hm) or dipyridyl (DIP). The cells were washed and diluted with PBS (starting at OD660 = 1.0). Varying dilutions of P. gingivalis cells were adsorbed on the wells of the microtiter plate and reacted with pre-immune serum (A) or purified pre-immune IgGs (pre) (B) and immune anti-HmuY serum (A) or purified immune anti-HmuY IgGs (im) (B). Representative data are shown. [file 1471-2180-10-134-S2.DOC]

**Additional file 2: Analysis of surface exposure of HmuY.** Analysis of surface exposure of *P. gingivalis* HmuY analyzed by whole-cell ELISA. *P. gingivalis* wild-type (A7436, W83) and *hmuY* deletion mutant (TO4) strains were grown in basal medium supplemented with hemin (Hm) or dipyridyl (DIP). The cells were washed and diluted with PBS (starting at OD_660_=1.0). Varying dilutions of *P. gingivalis* cells were adsorbed on the wells of the microtiter plate and reacted with pre-immune serum (A) or purified pre-immune IgGs (pre) (B) and immune anti-HmuY serum (A) or purified immune anti-HmuY IgGs (im) (B). Representative data are shown.
