# Supplementary material for: Species specificity, surface exposure, protein expression, immunogenicity, and participation in biofilm formation of Porphyromonas gingivalis HmuY
Source: BMC Microbiol. 2010 May 4;10:134. doi: 10.1186/1471-2180-10-134 (PMC2873494; doi:10.1186/1471-2180-10-134)
Supplement: Additional file 3 — P. gingivalis growth in broth cultures and biofilms, and biofilm accumulation. P. gingivalis growth was analyzed by measuring the OD at 660 nm, cell viability by plating cells on ABA plates and colony forming unit (CFU) calculation, and biofilm accumulation by microtiter plate assay. [file 1471-2180-10-134-S3.DOC]

**Additional file 3: *P. gingivalis* growth in broth cultures and biofilms, and biofilm accumulation.** *P. gingivalis* growth was analyzed by measuring the OD at 660 nm, cell viability by plating cells on ABA plates and colony forming unit (CFU) calculation, and biofilm accumulation by microtiter plate assay.

|  | Broth OD_660_/ CFU^a^ (n=7) | Biofilm OD_660_/CFU (n=3) | Biofilm A_570_ (n=24) | Broth OD_660_/CFU (n=7)^b^ | Biofilm OD_660_/CFU (n=3) | Biofilm A_570_ (n=24) |
| --- | --- | --- | --- | --- | --- | --- |
| 24 h | BM+Hm | | | BM+DIP | | |
| A7436 | 1.29±0.12/1.36±0.25 | 0.21±0.04/0.18±0.06 | 0.34±0.09 | 0.90±0.18/0.73±0.18 | 0.39±0.07/0.30±0.06 | 1.31±0.11 |
| TO4 | 1.23±0.15/1.29±0.19 | 0.15±0.03/0.13±0.06 | 0.27±0.08 | 0.83±0.11/0.77±0.23 | 0.19±0.05/0.18±0.04 | 0.54±0.10 |
| W83 | 1.35±0.14/1.41±0.21 | 0.26±0.06/0.24±0.04 | 0.35±0.11 | 0.82±0.15/0.80±0.29 | 0.48±0.07/0.41±0.07 | 1.38±0.11 |
| ATCC 33277 | 1.09±0.12/1.14±0.26 | 0.31±0.05/0.28±0.07 | 0.48±0.12 | 0.77±0.16/0.73±0.24 | 0.55±0.08/0.46±0.08 | 1.64±0.18 |
| 48 h | BM+Hm | | | BM+DIP | | |
| A7436 | 1.36±0.16/1.26±0.19 | 0.28±0.07/0.22±0.05 | 0.42±0.11 | 0.89±0.19/0.81±0.26 | 0.48±0.08/0.40±0.06 | 1.30±0.15 |
| TO4 | 1.22±0.14/1.11±0.22 | 0.18±0.05/0.17±0.06 | 0.40±0.08 | 0.85±0.11/0.81±0.19 | 0.21±0.06/0.17±0.05 | 0.69±0.10 |
| W83 | 1.35±0.14/1.29±0.19 | 0.33±0.08/0.31±0.04 | 0.46±0.11 | 0.81±0.14/0.80±0.24 | 0.55±0.07/0.48±0.08 | 1.62±0.18 |
| ATCC 33277 | 1.26±0.15/1.20±0.28 | 0.40±0.07/0.37±0.08 | 0.56±0.12 | 0.75±0.16/0.77±0.31 | 0.71±0.08/0.62±0.08 | 1.83±0.14 |

^a^ CFU, colony forming units are expressed as number of colonies×10^9^ per 1 ml

^b^ Bacteria analyzed after 1^st^ passage in basal medium without added hemin and supplemented with dipyridyl
